# Supplementary material for: Interaction between SNAI2 and MYOD enhances oncogenesis and suppresses differentiation in Fusion Negative Rhabdomyosarcoma
Source: Nat Commun. 2021 Jan 8;12:192. doi: 10.1038/s41467-020-20386-8 (PMC7794422; doi:10.1038/s41467-020-20386-8)
Supplement: Supplementary file 1 — Supplementary information [file 41467_2020_20386_MOESM1_ESM.pdf]

Figure S1

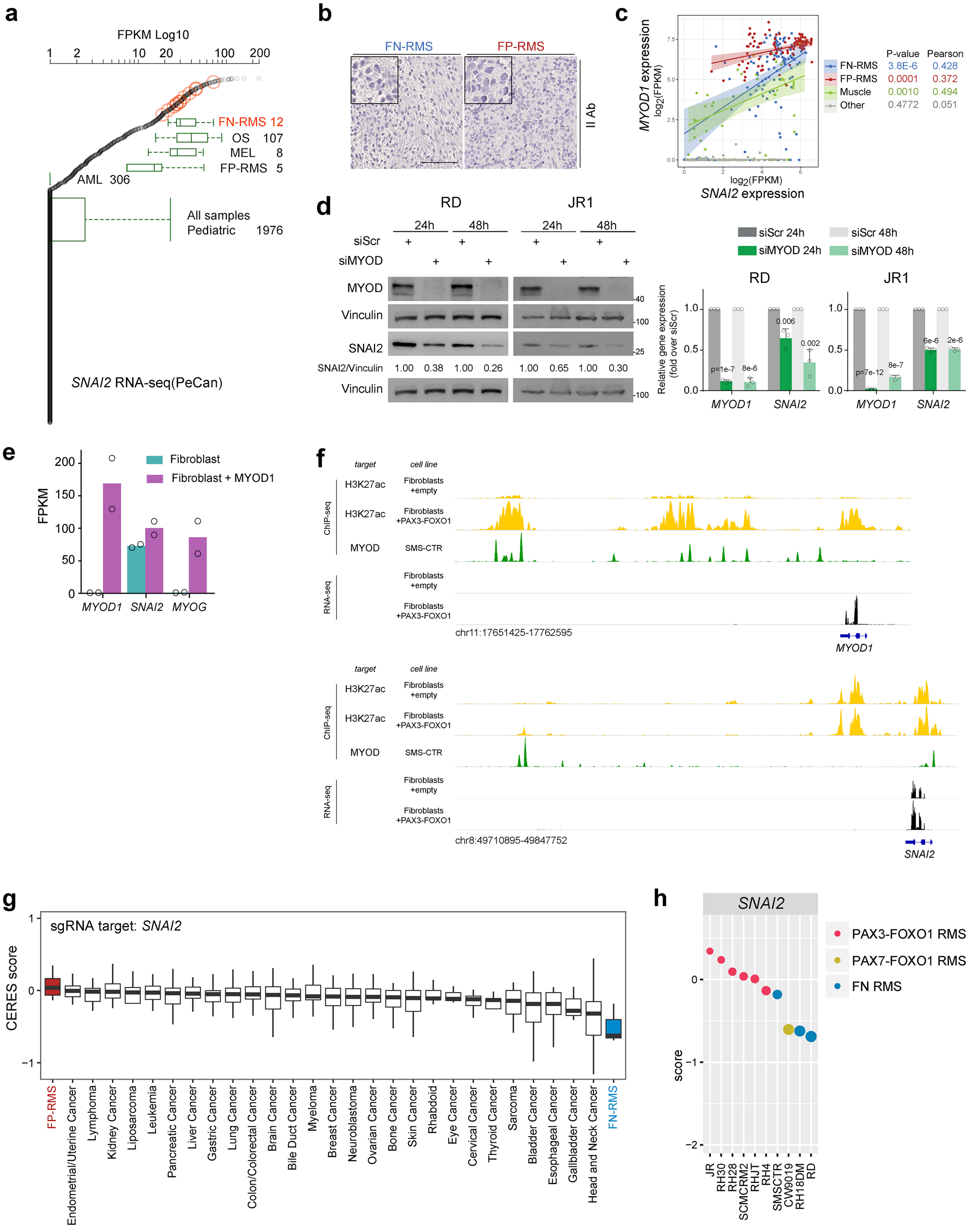
a
FPKM Log10
1 2 10 20 100 200
FN-RMS 12
OS 107
MEL 8
FP-RMS 5
AML 306
All samples
Pediatric 1976
SNAI2 RNA-seq(PeCan)
b
FN-RMS
FP-RMS
II Ab
c
MYOD1 expression
log2(FPKM)
log2(FPKM)
SNAI2 expression
P-value Pearson
FN-RMS 3.8E-6 0.428
FP-RMS 0.0001 0.372
Muscle 0.0010 0.494
Other 0.4772 0.051
d
RD
JR1
24h 48h 24h 48h
siScr
siMYOD
MYOD
Vinculin
SNAI2
SNAI2/Vinculin 1.00 0.38 1.00 0.26 1.00 0.65 1.00 0.30
Vinculin
siScr 24h
siScr 48h
siMYOD 24h
siMYOD 48h
Relative gene expression (fold over siScr)
MYOD1
SNAI2
e
FPKM
Fibroblast
Fibroblast + MYOD1
MYOD1 SNAI2 MYOG
f
target
cell line
ChIP-seq
RNA-seq
H3K27ac
MYOD
Fibroblasts +empty
Fibroblasts +PAX3-FOXO1
SMS-CTR
chr11:17651425-17762595
MYOD1
chr8:49710895-49847752
SNAI2
g
sgRNA target: SNAI2
CERES score
FP-RMS
Endometrial/Uterine Cancer
Lymphoma
Kidney Cancer
Liposarcoma
Leukemia
Pancreatic Cancer
Liver Cancer
Gastric Cancer
Lung Cancer
Colon/Colorectal Cancer
Brain Cancer
Bile Duct Cancer
Myeloma
Breast Cancer
Neuroblastoma
Ovarian Cancer
Bone Cancer
Skin Cancer
Rhabdoid
Eye Cancer
Cervical Cancer
Thyroid Cancer
Sarcoma
Bladder Cancer
Esophageal Cancer
Gallbladder Cancer
Head and Neck Cancer
FN-RMS
h
SNAI2
score
PAX3-FOXO1 RMS
PAX7-FOXO1 RMS
FN RMS
JR
RH30
RH28
SCMCRM2
RHJT
RH4
SMSCTR
CW9019
RH18DM
RD

Figure S1. Related to Figure1; *SNAI2* is highly expressed in RMS and is regulated by a MYOD bound super enhancer.

(a) Scatter plot for RNA-seq data of *SNAI2* expression in RMS (St.Jude PeCan database), expression in Melanoma, Osteosarcoma and AML is shown for comparison. (n=12 FN-RMS, n=5 FP-RMS, data represented as box and whiskers with mean +/- maxima)

(b) Representative images of the staining with secondary antibody as control, for RMS (n=6 independent experiments). Scale Bar = 100 μM.

(c) Pearson correlation analysis of *MYOD1* versus *SNAI2* in FN-RMS (n=108), FP-RMS (n=104) primary tumors, normal muscle (n=41) and other (n=197) tissues. P-value was calculated by a two tailed Pearson correlation test. Error bands represent the 95% confidence interval for the Loess curve fit of the data.

(d) A validated siRNA targeting *MYOD1* was used to knockdown MYOD expression in RD and JR1 cells. MYOD and SNAI2 expression was detected by western blot (left) and qRT-PCR (right). Data was normalized to cells treated with scramble siRNA. (n=3 biologically independent experiments, data presented as mean values +/- SD, Student's two tailed t-test, exact p values are reported in the figure).

(e) RNA-seq data of *MYOD1*, *SNAI2 and MYOG* expression in normal and *MYOD1*-overexpressed human primary skin fibroblasts (GSE93263). Bars show median (error bars = range) of 2 biologically independent experiments. FPKM, Fragments Per Kilobase of transcript per Million mapped reads.

(f) H3K27ac ChIP-seq and RNA-seq tracks, at *MYOD1* and *SNAI2* loci, shown for fibroblast (7250) cells with empty vector or with PAX3-FOXO1 cDNA. MYOD ChIP-seq data at the same loci in FN-RMS cell lines (SMS-CTR).

(g) Box plot comparing depletion of SNAI2 using CRISPR from Achilles project across different subtypes of cancers (https://depmap.org/portal/achilles/). Box plots show quartiles, black bar shows the median, and whiskers show the 1.5 × interquartile range. The number of the cell lines for each tumor type is reported on the DepMap Portal.

(h) Plot comparing depletion of SNAI2 using CRISPR from Achilles project across different RMS cell lines.

Figure S2

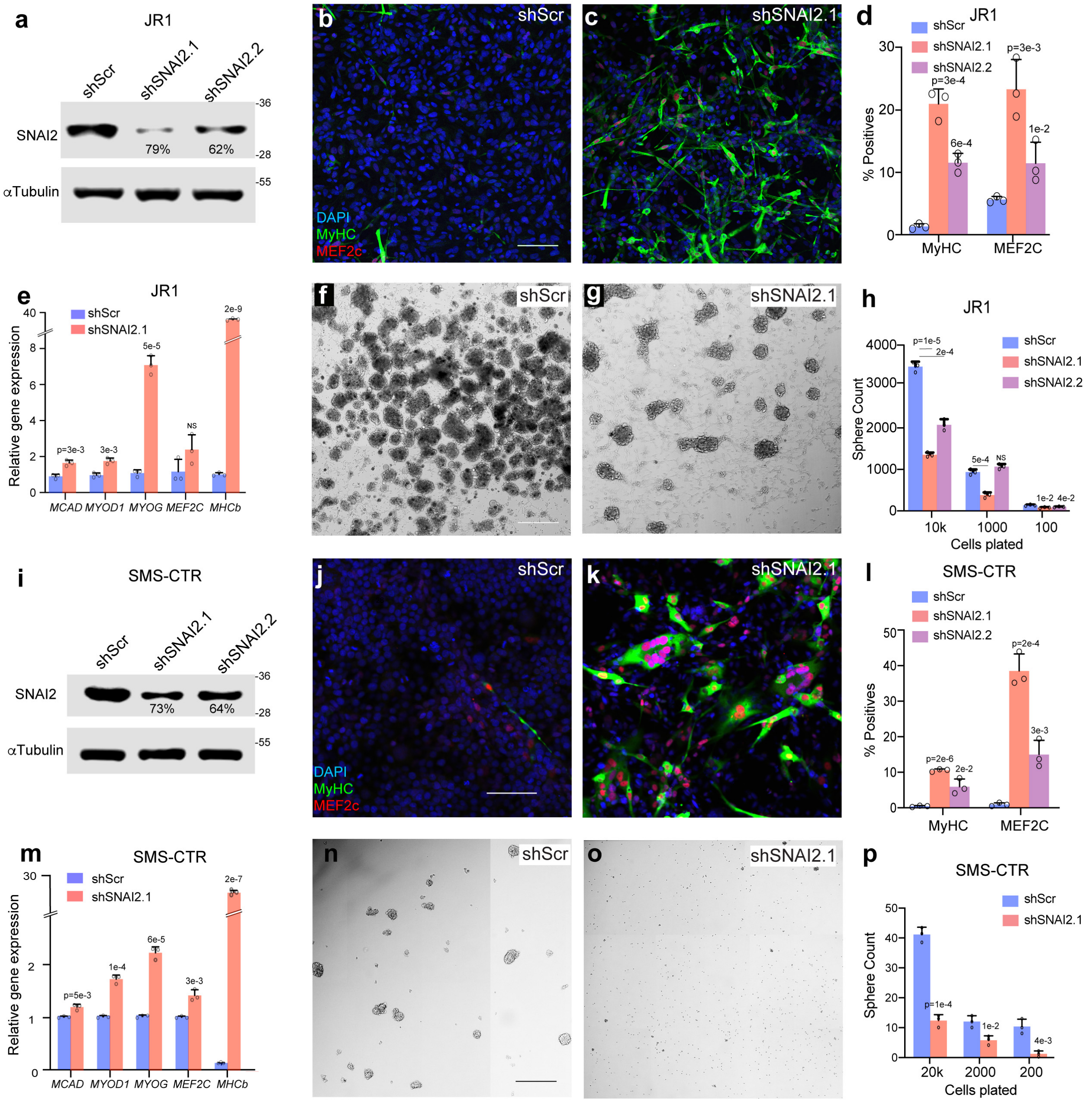
a
JR1
shScr
shSNAI2.1
shSNAI2.2
SNAI2
79%
62%
αTubulin
-36
-28
-55
b
shScr
c
shSNAI2.1
DAPI
MyHC
MEF2c
d
JR1
shScr
shSNAI2.1
shSNAI2.2
% Positives
MyHC
MEF2C
e
JR1
Relative gene expression
MCAD
MYOD1
MYOG
MEF2C
MHCb
f
g
h
Sphere Count
Cells plated
i
SMS-CTR
73%
64%
j
k
l
m
n
o
p

Figure S2 Related to Figure 2; Suppression of *SNAI2* activates myogenic differentiation and suppresses stemness *in vitro* in FN-RMS.

(a) Level of SNAI2 knockdown by shSNAI2.1 and shSNAI2.2 in JR1 cells compared to shScr assessed by western blot (Representative blot, n=3 biologically independent experiments).

(b-c) Representative images of immunostaining in JR1 cells stably expressing shScr or shSNAI2.1 shRNA stained for Myosin Heavy Chain 1 (MyHC, green), MEF2C (red) and DAPI for nuclei (blue).

(d) Quantitation of immunostaining counts as percentage value to total nuclei per image (n=3 biologically independent experiments, data presented as mean values +/- SD, Student's two tailed t-test, exact p values are reported in the figure).

(e) qRT-PCR gene expression analysis of JR1 cells comparing shScr to shSNAI2.1 showing early and late myogenic markers (n=3 biologically independent experiments, data presented as mean values +/- SD, Student's two tailed t-test, exact p values are reported in the figure).

(f-g) Representative images of sphere formation assay, in JR1 cells containing shScr or shSNAI2.1.

(h) Quantitation of sphere counts in JR1 cells plated at three densities (10,000, 1,000 and 100 per well (n=3 biologically independent experiments, data presented as mean values +/- SD, Student's two tailed t-test, exact p values are reported in the figure).

(i) Level of SNAI2 knockdown by shSNAI2.1 and shSNAI2.2 in SMS-CTR cells compared to shScr assessed by western blot (Representative blot, n =3 biologically independent experiments).

(j-k) Representative images of immunostaining of SMS-CTR cells stably expressing shScr or shSNAI2.1 shRNA stained for Myosin Heavy Chain 1 (MyHC, green), MEF2C (red) and DAPI for nuclei (blue).

(l) Quantitation of immunostaining counts as percentage value to total nuclei per image (n=3 biologically independent experiments, data presented as mean values +/- SD, Student's two tailed t-test, exact p values are reported in the figure).

(m) qRT-PCR gene expression analysis of SMS-CTR cells comparing shScr to shSNAI2.1 showing early and late myogenic markers (n=3 biologically independent experiments, data presented as mean values +/- SD, Student's two tailed t-test, exact p values are reported in the figure).

(n-o) Representative images of sphere formation assay in SMS-CTR cells containing shScr or shSNAI2.1.

(p) Quantitation of sphere counts in SMS-CTR cells plated at three densities (20,000, 2000 and 200 per well) (n=3 biologically independent experiments, data presented as mean values +/- SD, Student’s two tailed t-test, exact p values are reported in the figure), Scale Bars in b, f, j, n = 100 μM.

Figure S3

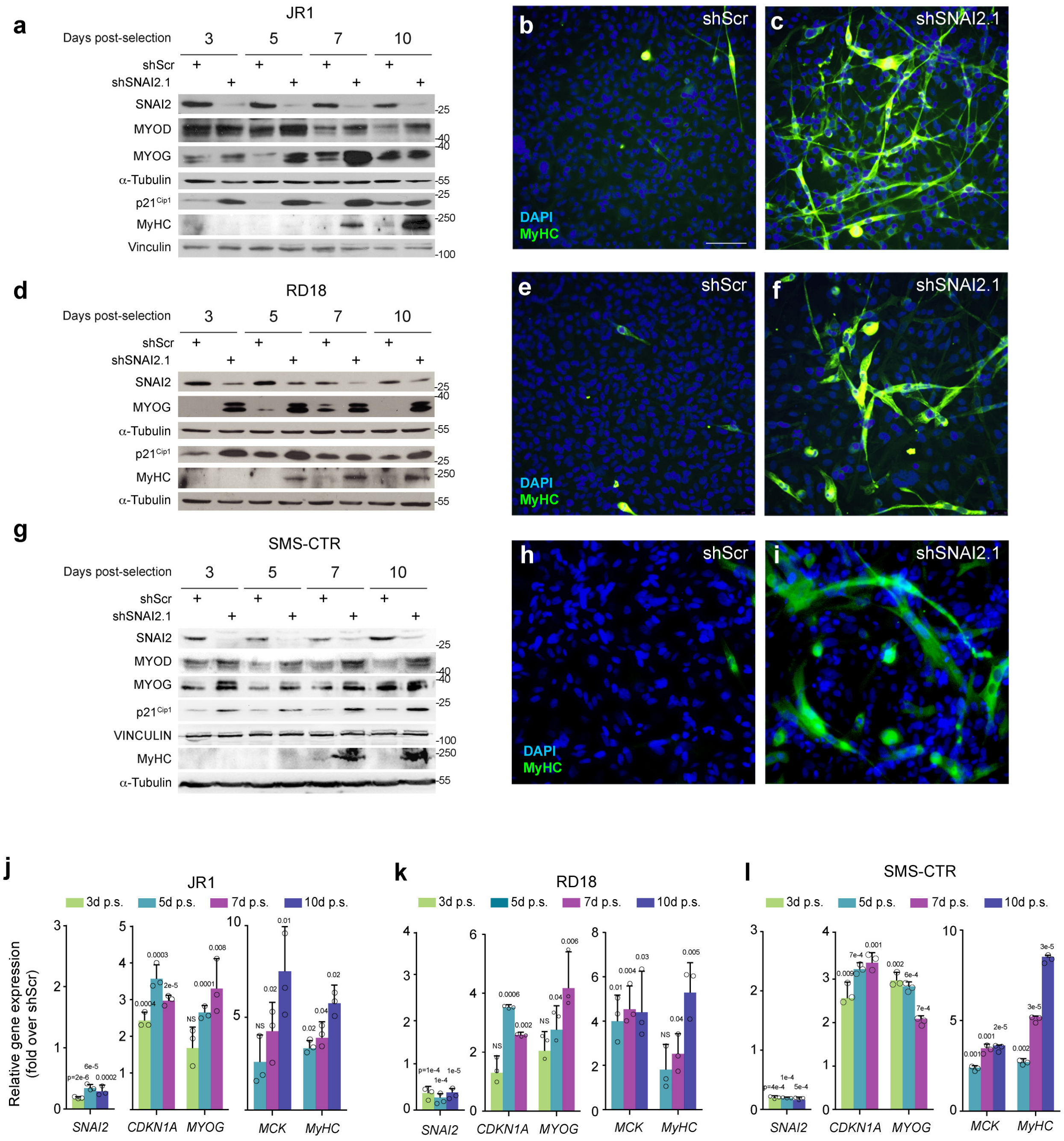
a
JR1
Days post-selection
3
5
7
10
shScr
shSNAI2.1
SNAI2
MYOD
MYOG
α-Tubulin
p21 Cip1
MyHC
Vinculin
b
shScr
DAPI
MyHC
c
shSNAI2.1
d
RD18
e
f
g
SMS-CTR
VINCULIN
h
i
j
k
l
3d p.s.
5d p.s.
7d p.s.
10d p.s.
Relative gene expression
(fold over shScr)
SNAI2
CDKN1A
MYOG
MCK
MyHC

Figure S3. Related to Figure 2; Suppression of *SNAI2* activates myogenic differentiation and suppresses stemness *in vitro* in FN-RMS.

(a,d,g) Representative western blot (n=3 biologically independent experiments) showing muscle differentiation markers in SNAI2 knockdown for JR1, RD18 and SMS-CTR cells respectively, at different time points post puromycin selection in growth medium.

(b, c, e, f, h, i) Representative immunostaining (n=3 biologically independent experiments) of short-term JR1, RD18 and SMS-CTR cells respectively, expressing shScr or shSNAI2.1 shRNA stained for Myosin Heavy Chain 1 (MyHC, green) and DAPI for nuclei (blue). Scale Bar in b = 100 μM.

(j-l) Gene expression analysis in JR1, RD18 and SMS-CTR cells respectively, comparing shScr to shSNAI2.1 showing early and late myogenic markers. (n=3 biologically independent experiments, data presented as mean values +/- SD, Student's two tailed t-test, exact p values are reported in the figure, NS not significant).

Figure S4

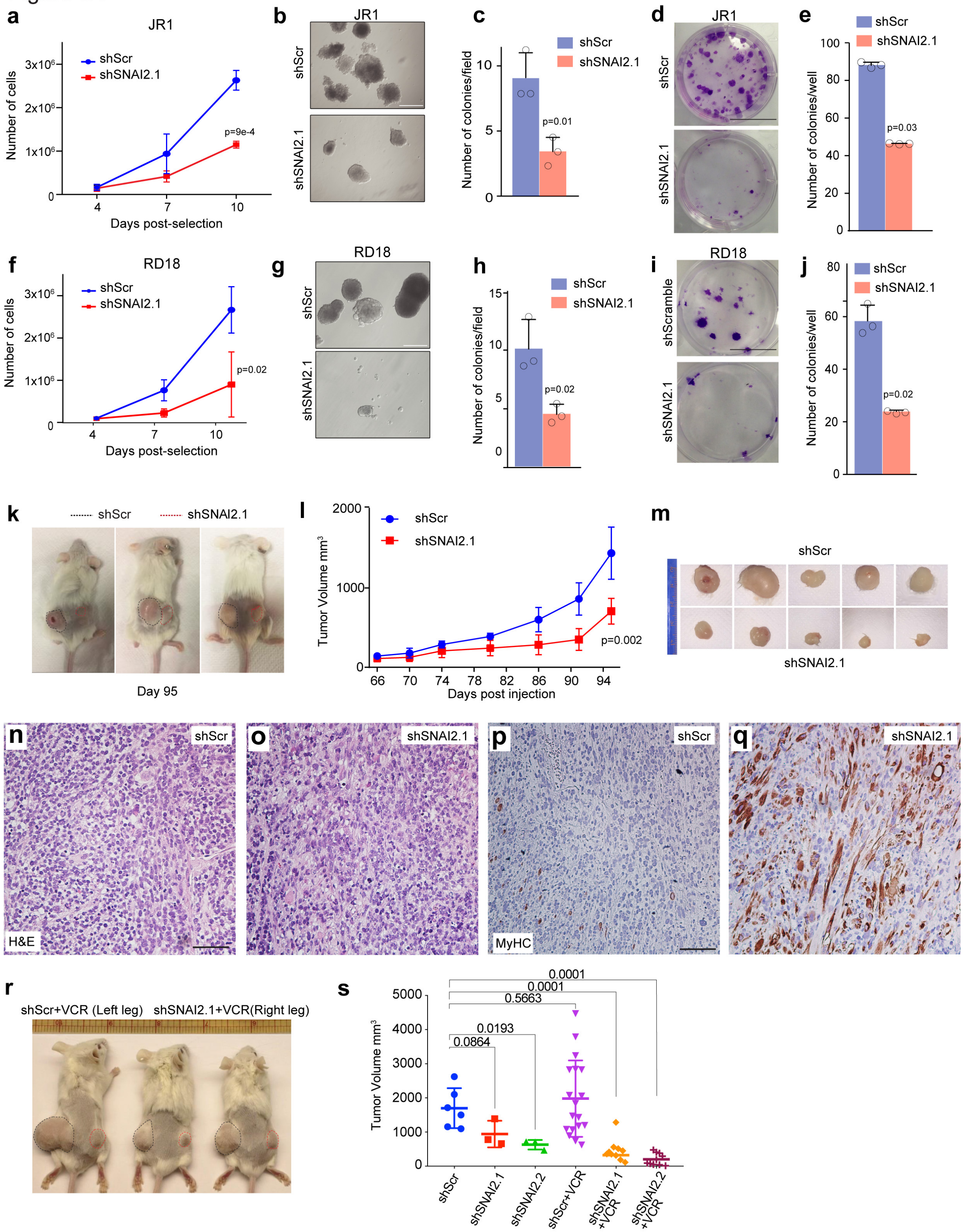

a
JR1
Number of cells
3x10⁶
2x10⁶
1x10⁶
0
shScr
shSNAI2.1
p=9e-4
4
7
10
Days post-selection
b
JR1
shScr
shSNAI2.1
c
Number of colonies/field
shScr
shSNAI2.1
p=0.01
d
JR1
shScr
shSNAI2.1
e
shScr
shSNAI2.1
Number of colonies/well
p=0.03
f
RD18
Number of cells
shScr
shSNAI2.1
p=0.02
Days post-selection
g
RD18
shScr
shSNAI2.1
h
shScr
shSNAI2.1
Number of colonies/field
p=0.02
i
RD18
shScramble
shSNAI2.1
j
shScr
shSNAI2.1
Number of colonies/well
p=0.02
k
shScr
shSNAI2.1
Day 95
l
shScr
shSNAI2.1
Tumor Volume mm3
p=0.002
Days post injection
m
shScr
shSNAI2.1
n
shScr
H&E
o
shSNAI2.1
p
shScr
MyHC
q
shSNAI2.1
r
shScr+VCR (Left leg)
shSNAI2.1+VCR(Right leg)
s
Tumor Volume mm3
0.0001
0.0001
0.5663
0.0193
0.0864
shScr
shSNAI2.1
shSNAI2.2
shScr+VCR
shSNAI2.1 +VCR
shSNAI2.2 +VCR

Figure S4. Related to Figure 3; Suppression of *SNAI2* reduces tumorigenicity and growth, and induces muscle differentiation *in vitro and in vivo* in FN-RMS; which is enhanced by vincristine

(a) Growth curve analysis of JR1 cells three days post puromycin selection after lentiviral infection with shScr or shSNAI2 shRNAs. (n=3 biologically independent experiments, data presented as mean values +/- SD, Student's two tailed t-test, exact p values are reported in the figure).

(b,c) Representative images of soft agar assay comparing shSNAI2 infected JR1 cells to shScr cells, quantitation values of colonies as bar graph. (n=3 biologically independent experiments, data presented as mean values +/- SD, Student's two tailed t-test, exact p values are reported in the figure). Scale Bar in b = 200μM.

(d,e) Representative images of colony formation assay with JR1 cells and quantification of colonies. (n=3 biologically independent experiments, data presented as mean values +/- SD, Student's two tailed t-test, exact p values are reported in the figure). Scale Bar in d = 10mm.

(f) Growth curve analysis of RD18 cells three days post puromycin selection after lentiviral infection with shScr or shSNAI2 shRNAs. (n=3 biologically independent experiments, data presented as mean values +/- SD, Student's two tailed t-test, exact p values are reported in the figure).

(g,h) Representative images of soft agar assay comparing shSNAI2 infected RD18 cells to shScr shRNA cells, quantitation values of colonies as bar graph. (n=3 biologically independent experiments, data presented as mean values +/- SD, Student's two tailed t-test, exact p values are reported in the figure). Scale Bars = 200μM.

(i,j) Representative images of colony formation assay in RD18 cells and quantification of colonies. (n=3 biologically independent experiments, data presented as mean values +/- SD, Student's two tailed t-test, exact p values are reported in the figure). Scale Bar in i = 10mm.

(k) *In vivo* assessment of tumor forming ability of shSNAI2 JR1 cells. Mice were injected with JR1 cells (shScr- Left, shSNAI2.2- Right, $1x10^6$cells) and measured weekly for 95 days.

(l) Tumor volume of mice injected with either shScr or shSNAI2 assessed weekly by caliper measurement represented as mm^3 (n=5 mice, data presented as mean values +/- SD, Student's two tailed t-test, exact p values are reported in the figure).

(m) Images of shScr and shSNAI2.2 JR1 tumors from injected mice.

(n-q) Representative immunohistochemistry (n=5) of JR1 cell xenografts after 95 days of growth in mice, Hematoxylin and Eosin (H&E) and MyHC staining in the same tumors.

(r) Tumor growth of RD cell xenografts in mice with shScr or shSNAI2.2 tumors in left and right flank respectively compared to shScr and shSNAI2.2 with vincristine (VCR) treatment.

(s) Tumor size measurement of xenograft tumors with either shScr or shSNAI2.2 and vincristine (VCR) treatment, post mortem (n=6 shScr, 3 shSNAI2.1, 3 shSNAI2.2, n= 20 shScr+VCR, 10 shSNAI2.1+VCR and 10 shSNAI2.2+VCR tumors each biologically independent experiments, data presented as mean values +/- SD, Student's two tailed t-test, exact p values are reported in the figure). Scale Bars in n, p= 100μM.

Figure S5

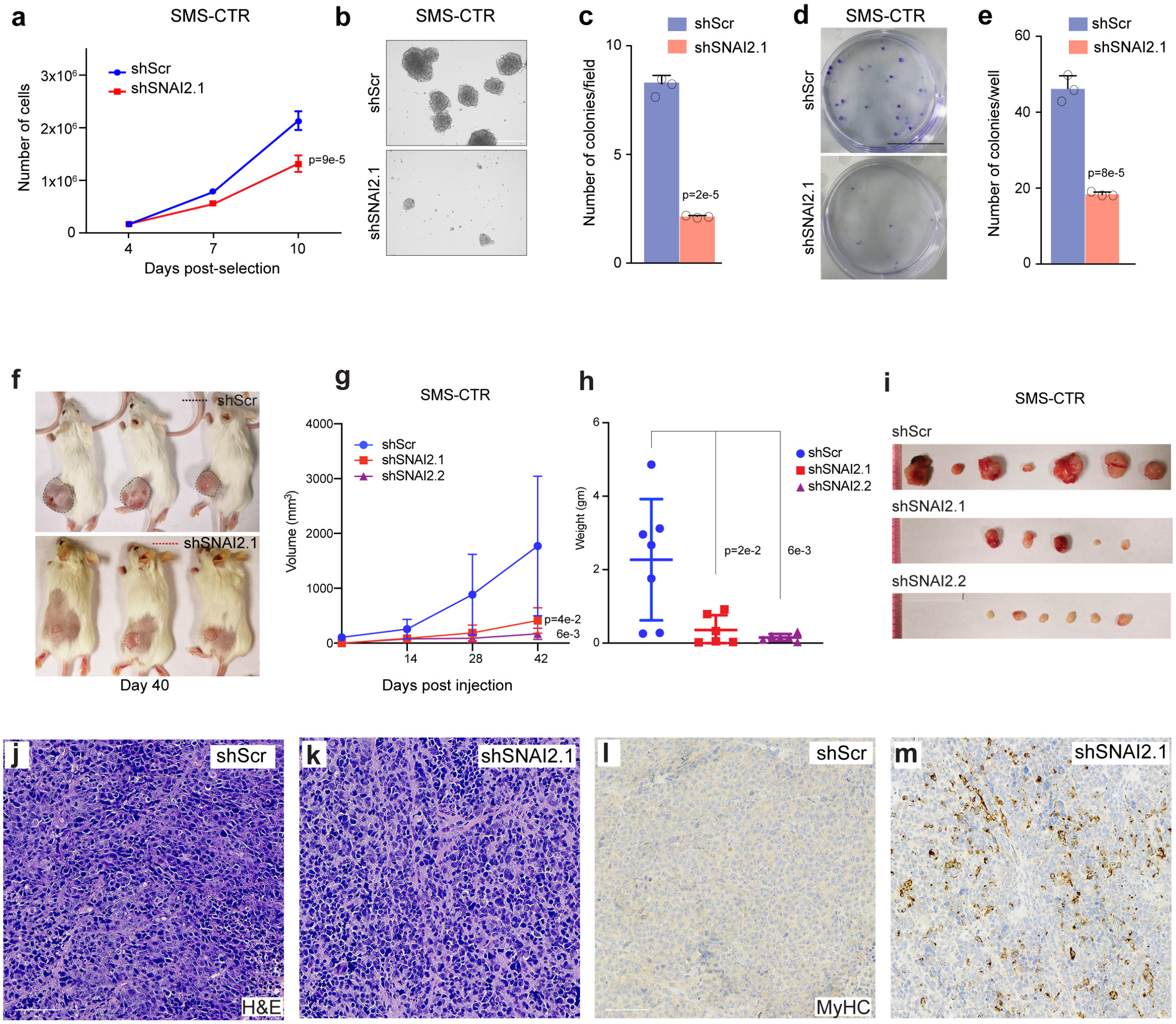

a
SMS-CTR
Number of cells
3x10⁶
2x10⁶
1x10⁶
0
shScr
shSNAI2.1
p=9e-5
4
7
10
Days post-selection
b
SMS-CTR
shScr
shSNAI2.1
c
Number of colonies/field
10
5
0
shScr
shSNAI2.1
p=2e-5
d
SMS-CTR
shScr
shSNAI2.1
e
Number of colonies/well
60
40
20
0
shScr
shSNAI2.1
p=8e-5
f
shScr
shSNAI2.1
Day 40
g
SMS-CTR
Volume (mm³)
4000
3000
2000
1000
0
shScr
shSNAI2.1
shSNAI2.2
p=4e-2
6e-3
14
28
42
Days post injection
h
Weight (gm)
6
4
2
0
shScr
shSNAI2.1
shSNAI2.2
p=2e-2
6e-3
i
SMS-CTR
shScr
shSNAI2.1
shSNAI2.2
j
shScr
H&E
k
shSNAI2.1
l
shScr
MyHC
m
shSNAI2.1

Figure S5 Related to Figure 3; Suppression of *SNAI2* reduces tumorigenicity and growth, and induces muscle differentiation *in vitro and in vivo* in FN-RMS; which is enhanced by vincristine

(a) Growth curve analysis of SMS-CTR cells three days post puromycin selection after lentiviral infection with shScr or shSNAI2 shRNAs. (n=3 biologically independent experiments, data presented as mean values +/- SD, Student's two tailed t-test, exact p values are reported in the figure).

(b,c) Representative images of soft agar assay comparing shSNAI2 infected SMS-CTR cells to shScr cells, quantitation values of colonies as bar graph. (n=3 biologically independent experiments, data presented as mean values +/- SD, Student's two tailed t-test, exact p values are reported in the figure). Scale Bars = 200μM.

(d,e) Representative images of colony formation assay with SMS-CTR cells and quantification of colonies. (n=3 biologically independent experiments, data presented as mean values +/- SD, Student's two tailed t-test, exact p values are reported in the figure). Scale bar in d = 10 mm.

(f) *In vivo* assessment of tumor forming ability comparing shScr control to shSNAI2 SMS-CTR cells. Mice were injected with SMS-CTR cells (shScr- Top, shSNAI2.1 Bottom, $5x10^6$ cells) and measured weekly for 42 days.

(g,h) Tumor volume of mice injected with either shScr or shSNAI2 assessed weekly by caliper measurement represented as mm^3. Weight measurement of mice tumors with either shScr or shSNAI2.1, SNAI2.2 post mortem (n= 7 shScr, 6 shSNAI2.1 and 6 shSNAI2.2 tumors each biologically independent experiments, data presented as mean values +/- SD, Student's two tailed t-test, exact p values are reported in the figure).

(i) Images of shScr and shSNAI2.2 SMS-CTR tumors from injected mice.

(j-m) Immunohistochemistry of SMS-CTR cell xenografts after 42 days of growth in mice, Hematoxylin and Eosin (H&E) and MyHC staining in the same tumors. Scale Bars in j, l = 100μM.

Figure S6

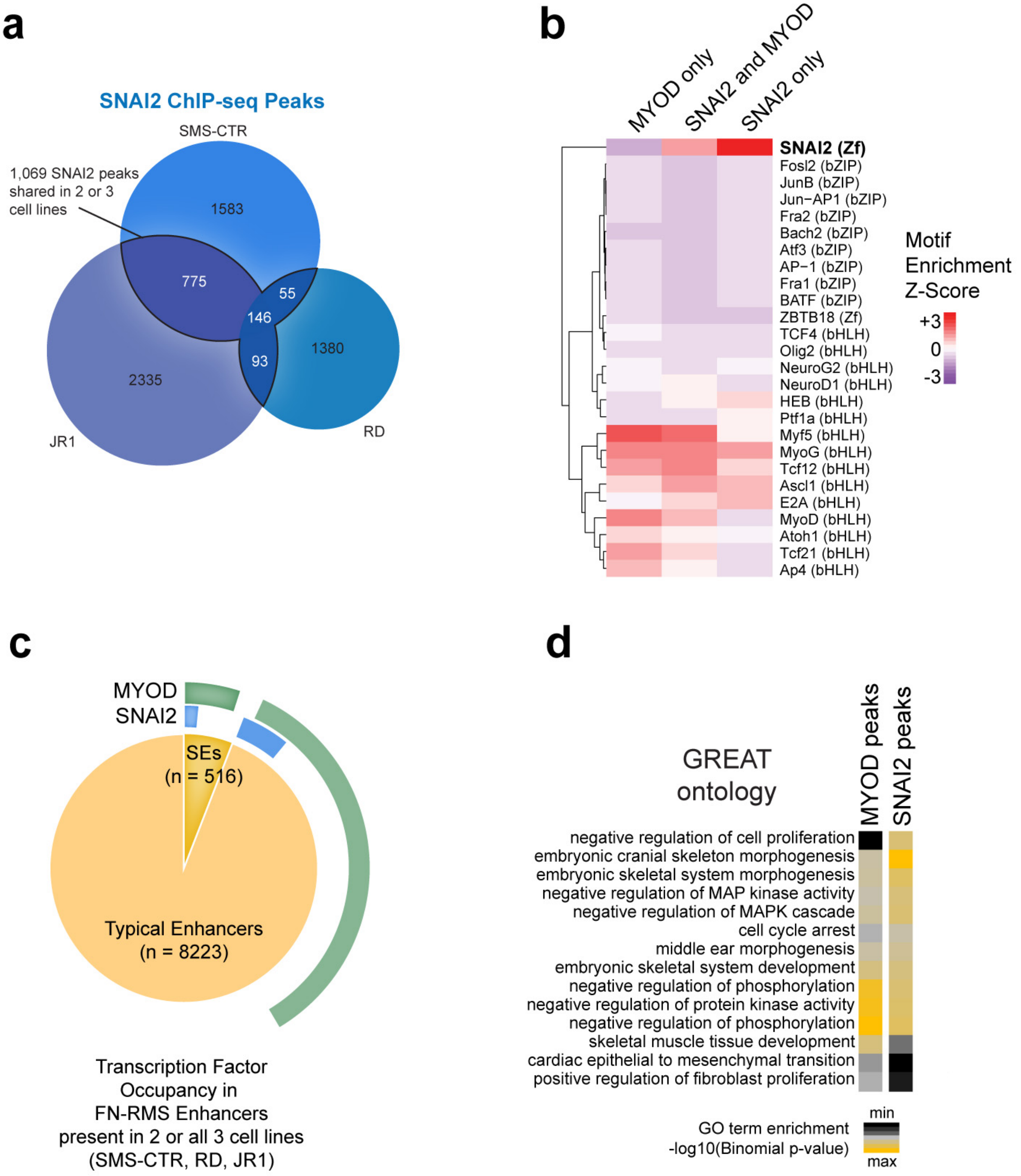
a
SNAI2 ChIP-seq Peaks
SMS-CTR
1,069 SNAI2 peaks shared in 2 or 3 cell lines
1583
775
55
146
93
1380
2335
JR1
RD
b
MYOD only
SNAI2 and MYOD
SNAI2 only
SNAI2 (Zf)
Fosl2 (bZIP)
JunB (bZIP)
Jun−AP1 (bZIP)
Fra2 (bZIP)
Bach2 (bZIP)
Atf3 (bZIP)
AP−1 (bZIP)
Fra1 (bZIP)
BATF (bZIP)
ZBTB18 (Zf)
TCF4 (bHLH)
Olig2 (bHLH)
NeuroG2 (bHLH)
NeuroD1 (bHLH)
HEB (bHLH)
Ptf1a (bHLH)
Myf5 (bHLH)
MyoG (bHLH)
Tcf12 (bHLH)
Ascl1 (bHLH)
E2A (bHLH)
MyoD (bHLH)
Atoh1 (bHLH)
Tcf21 (bHLH)
Ap4 (bHLH)
Motif Enrichment Z-Score
+3
0
-3
c
MYOD
SNAI2
SEs (n = 516)
Typical Enhancers (n = 8223)
Transcription Factor Occupancy in FN-RMS Enhancers present in 2 or all 3 cell lines (SMS-CTR, RD, JR1)
d
GREAT ontology
MYOD peaks
SNAI2 peaks
negative regulation of cell proliferation
embryonic cranial skeleton morphogenesis
embryonic skeletal system morphogenesis
negative regulation of MAP kinase activity
negative regulation of MAPK cascade
cell cycle arrest
middle ear morphogenesis
embryonic skeletal system development
negative regulation of phosphorylation
negative regulation of protein kinase activity
negative regulation of phosphorylation
skeletal muscle tissue development
cardiac epithelial to mesenchymal transition
positive regulation of fibroblast proliferation
GO term enrichment -log10(Binomial p-value)
min
max

Figure S6. Related Figure 4; SNAI2 binds key enhancers in FN-RMS

(a) Consistency of SNAI2 peaks interrogated in 3 FN-RMS cell lines (SMS-CTR, RD and JR1). Overlap was found in at least 2 of 3 cells for 1069 peaks (p=assigned by MACS2 peak calling algorithm).
(b) Sequence recognition motifs identified binding motifs found in MYOD only, SNAI2 and MYOD or SNAI2 only.
(c) SNAI2 and MYOD occupancy which occur in typical and super enhancers in FN-RMS present in 2 or all 3 cell lines.
(d) Gene ontology enrichments (assigned by GREAT) for MYOD and SNAI2 bound regions.

Figure S7

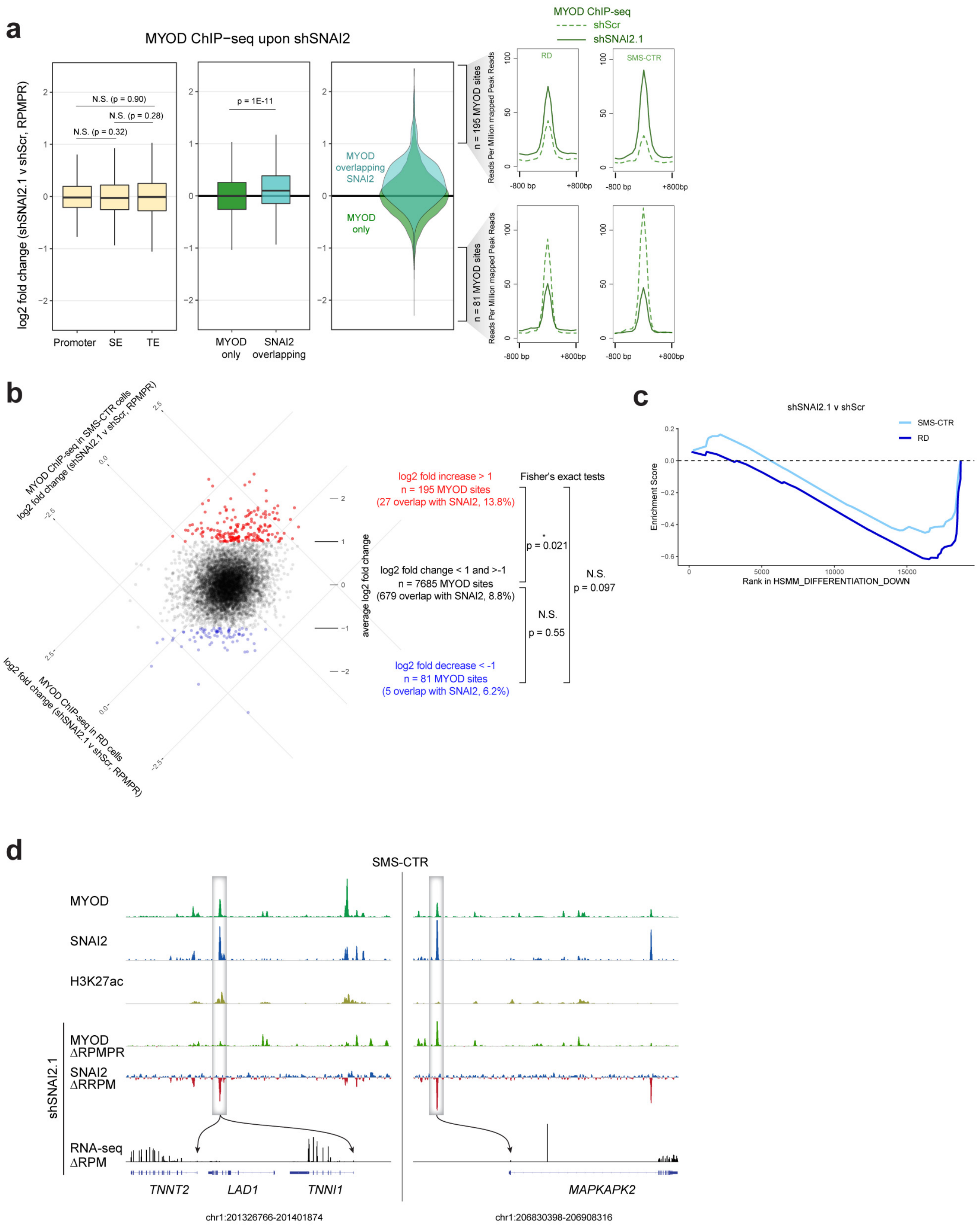

a
MYOD ChIP−seq upon shSNAI2
log2 fold change (shSNAI2.1 v shScr, RPMPR)
N.S. (p = 0.90)
N.S. (p = 0.28)
N.S. (p = 0.32)
Promoter
SE
TE
p = 1E-11
MYOD only
SNAI2 overlapping
MYOD overlapping SNAI2
MYOD only
MYOD ChIP-seq
shScr
shSNAI2.1
n = 195 MYOD sites
n = 81 MYOD sites
Reads Per Million mapped Peak Reads
RD
SMS-CTR
-800 bp
+800bp
b
MYOD ChIP-seq in SMS-CTR cells log2 fold change (shSNAI2.1 v shScr, RPMPR)
MYOD ChIP-seq in RD cells log2 fold change (shSNAI2.1 v shScr, RPMPR)
average log2 fold change
log2 fold increase > 1
n = 195 MYOD sites
(27 overlap with SNAI2, 13.8%)
log2 fold change < 1 and >-1
n = 7685 MYOD sites
(679 overlap with SNAI2, 8.8%)
log2 fold decrease < -1
n = 81 MYOD sites
(5 overlap with SNAI2, 6.2%)
Fisher's exact tests
*
p = 0.021
N.S.
p = 0.55
N.S.
p = 0.097
c
shSNAI2.1 v shScr
SMS-CTR
RD
Enrichment Score
Rank in HSMM_DIFFERENTIATION_DOWN
d
SMS-CTR
MYOD
SNAI2
H3K27ac
MYOD ΔRPMPR
SNAI2 ΔRRPM
RNA-seq ΔRPM
shSNAI2.1
TNNT2
LAD1
TNNI1
MAPKAPK2
chr1:201326766-201401874
chr1:206830398-206908316

Figure S7. Related to Figure 5; Ablation of SNAI2 enables MYOD to activate myogenic target genes.

(a) MYOD ChIP-seq peak signal intensity fold changes upon shSNAI2 at Promoter, SE and TE (left) and at MYOD unique or MYOD-SNAI2 overlapping regions (middle) visualized as box and violin plots. Composite plots showing increased (right, top) and decreased (right, bottom) MYOD signal intensities at MYOD-SNAI2 overlapping regions in SMS-CTR and RD. RPMPR, Reads Per Million Mapped Peak Reads; N.S., not significant; SE, Super Enhancer; TE, Typical Enhancer. Box plots show quartiles, black bar shows the median, and whiskers show the 1.5 × interquartile range. P values were evaluated using student's T test with Welch's correction.

(b) Scatter plot of significantly modulated MYOD peaks in shSNAI2-treated SMS-CTR (up, left) and RD (bottom, left). Red dots represent peaks with significantly increased enrichment in both SMS-CTR and RD (fold change > 2). Blue dots represent peaks without significant changes. N.S., not significant. Fischer's exact test was employed to compare indicated groups and exact p values are reported on the panel.

(c) Gene set enrichment analysis (GSEA) enrichment plots showing negative enrichment for a set of genes down-regulated during differentiation of human skeletal muscle myoblasts into myotubes. The false discovery rate (FDR) q value and the nominal P value is < 0.05.

(d)Sites of direct SNAI2 mediated gene suppression at *TNNT2*, *TNNI1* (left) and *MAPKAPK2* (right), with ChIP-seq. Representative ChIP-seq tracks are shown for MYOD, SNAI2, H3K27ac, and delta (Δ) value (shSNAI2.1 minus shScr) in MYOD and SNAI2 and gene expression (RNA-seq) at *TNNT2*, *TNNI1* and *MAPKAPK2* loci in SMS-CTR. Arrows depict SNAI2/MYOD regulation on direct target myogenic genes. RPMPR, Reads Per Million Mapped Peak Reads; RRPM, Reference-adjusted Reads Per Million Mapped Reads; RPM, Reads Per Million Mapped Reads.

Figure S8

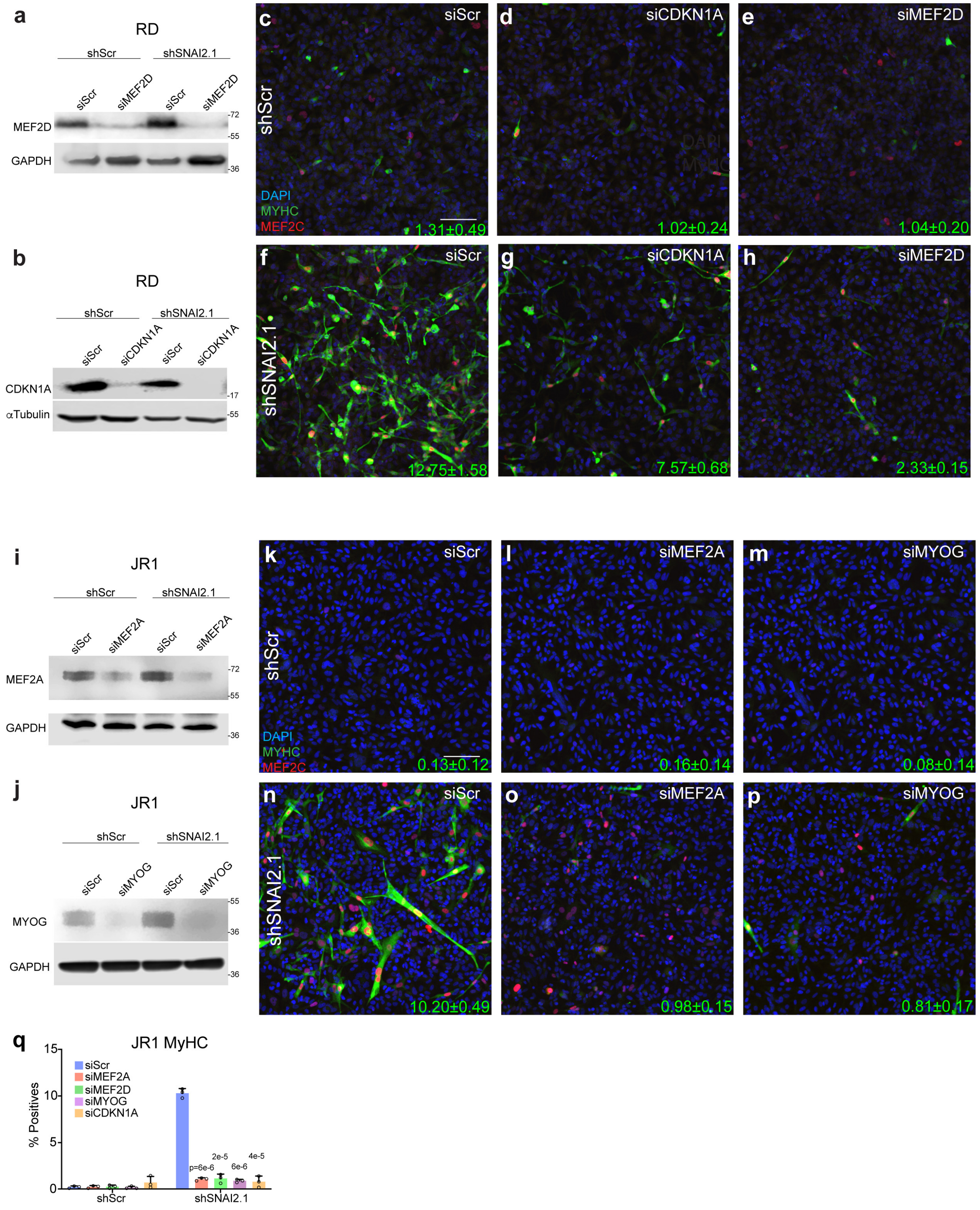

a
RD
shScr
shSNAI2.1
siScr
siMEF2D
siScr
siMEF2D
MEF2D
GAPDH
-72
-55
-36
b
RD
shScr
shSNAI2.1
siScr
siCDKN1A
siScr
siCDKN1A
CDKN1A
αTubulin
-17
-55
c
siScr
d
siCDKN1A
e
siMEF2D
shScr
DAPI
MYHC
MEF2C
1.31±0.49
1.02±0.24
1.04±0.20
f
siScr
g
siCDKN1A
h
siMEF2D
shSNAI2.1
12.75±1.58
7.57±0.68
2.33±0.15
i
JR1
shScr
shSNAI2.1
siScr
siMEF2A
siScr
siMEF2A
MEF2A
GAPDH
-72
-55
-36
j
JR1
shScr
shSNAI2.1
siScr
siMYOG
siScr
siMYOG
MYOG
GAPDH
-55
-36
-36
k
siScr
l
siMEF2A
m
siMYOG
shScr
DAPI
MYHC
MEF2C
0.13±0.12
0.16±0.14
0.08±0.14
n
siScr
o
siMEF2A
p
siMYOG
shSNAI2.1
10.20±0.49
0.98±0.15
0.81±0.17
q
JR1 MyHC
% Positives
15
10
5
0
siScr
siMEF2A
siMEF2D
siMYOG
siCDKN1A
p=6e-6
2e-5
6e-6
4e-5
shScr
shSNAI2.1

Figure S8. Related to Figure 6; *MYOG, MEF2A/D and CDKN1A* block differentiation downstream of *SNAI2*.

(a,b) Western blot of shScr and shSNAI2.1 RD cells transfected with siRNAs against MEF2D, CDKN1A, along with Control siRNA (scramble) (Representative blot, n =3 biologically independent experiments).
(c-h) MyHC and MEF2C immunostaining of shScr and shSNAI2 RD cells transfected with siRNA. (Representative images of n =3 biologically independent experiments).
(i-j) Western blot of shScr and shSNAI2.1 JR1 cells transfected with siRNAs against MEF2A, MYOG along with Control siRNA (scramble). (Representative blot, n =3 biologically independent experiments).
(k-p) MyHC and MEF2C immunostaining of shScr and shSNAI2 JR1 cells transfected with siRNAs. (Representative images of n =3 biologically independent experiments).
(q) Quantification MyHC staining in JR1 shScr and shSNAI2.1 cells transfected with different siRNAs, data averaged over images from three replicates. (n=3 biologically independent experiments, data presented as mean values +/- SD, Student’s two tailed t-test, exact p values are reported in the figure). Scale Bars in c, k = 100μM.

Supplemental Table1

UP_RMS_Three_cell_lines	Indirect_UP_shSNAI2	Direct_UP_SNAI2 Targets
ACTA1	ACTA1	ACTC1
ACTC1	ACTN2	ACVR2A
ACTN2	AGL	ADAMTS14
ACVR2A	ANKRD2	AFAP1L1
ADAMTS14	APOBEC2	ALDH1B1
AFAP1L1	AQP1	ARPP21
AGL	ATP2A1	CAMK2B
ALDH1B1	BLCAP	FAM212B
ANKRD2	CACNA1S	FAM65B
APOBEC2	CACNG1	FNDC5
AQP1	CARNS1	KRT80
ARPP21	CASQ2	MAPKAPK2
ATP2A1	CASZ1	MEF2A
BLCAP	CAV3	MYBPH
CACNA1S	CCDC141	MYOG
CACNG1	CD36	MYOM1
CAMK2B	CDKN1A	PVALB
CARNS1	CHRNB1	RAPGEF1
CASQ2	CKB	RASSF4
CASZ1	CKM	REEP1
CAV3	COL5A3	RYR1
CCDC141	COX6A2	SIRT2
CD36	CSRP3	SORBS2
CDKN1A	CYB5R1	SVIL
CHRNB1	DCLK1	TNNI1
CKB	DMPK	TNNT2
CKM	DYSF	TSPAN33
COL5A3	EGLN3	USP2
COX6A2	ENO3	WIPF3
CSRP3	ERBB3	
CYB5R1	F13A1	
DCLK1	FABP3	
DMPK	FBXO32	
DYSF	FOLR1	
EGLN3	GADD45G	

ENO3	GMPR
ERBB3	HBEGF
F13A1	HDAC9
FABP3	HES6
FAM212B	HFE2
FAM65B	HRC
FBXO32	HSPB3
FNDC5	ITGA7
FOLR1	KISS1
GADD45G	KLHL40
GMPR	KLHL41
HBEGF	LAD1
HDAC9	LDB3
HES6	LMOD3
HFE2	MAPRE3
HRC	MB
HSPB3	MEF2C
ITGA7	MRAS
KISS1	MURC
KLHL40	MYH3
KLHL41	MYH7
KRT80	MYH8
LAD1	MYL1
LDB3	MYL4
LMOD3	MYL5
MAPKAPK2	MYL6B
MAPRE3	MYLPF
MB	MYOM2
MEF2A	MYOM3
MEF2C	MYOZ1
MRAS	MYOZ2
MURC	NEB
MYBPH	NPNT
MYH3	OBSCN
MYH7	PADI2
MYH8	PDE4DIP
MYL1	PGAM2
MYL4	PRNP
MYL5	PSEN2

MYL6B	RB1
MYLPF	SEMA6B
MYOG	SH3BGR
MYOM1	SHD
MYOM2	SLC1A4
MYOM3	SLC7A7
MYOZ1	SLN
MYOZ2	SMPX
NEB	SMYD1
NPNT	SORBS1
OBSCN	SRL
PADI2	SRPK3
PDE4DIP	SYNPO2L
PGAM2	TMEM8C
PRNP	TMOD1
PSEN2	TNNC1
PVALB	TNNC2
RAPGEF1	TNNI2
RASSF4	TNNT3
RB1	TPM1
REEP1	TRIM54
RYR1	TTN
SEMA6B	UCP2
SH3BGR	UNC45B
SHD	ZNF106
SIRT2	
SLC1A4	
SLC7A7	
SLN	
SMPX	
SMYD1	
SORBS1	
SORBS2	
SRL	
SRPK3	
SVIL	
SYNPO2L	
TMEM8C	
TMOD1	

TNNC1	
TNNC2	
TNNI1	
TNNI2	
TNNT2	
TNNT3	
TPM1	
TRIM54	
TSPAN33	
TTN	
UCP2	
UNC45B	
USP2	
WIPF3	
ZNF106	

Supplemental Table2 (Key resources)

REAGENT/RESOURCE	SOURCE	IDENTIFIER
**Antibodies**		
Slug (C19G7) (1:800 WB; 10μg ChIP; 1:100 IHC)	CST	Cat# 9585, RRID:AB_2239535
Myosin Heavy Chain, (1:50 WB; 2μg/ml IF; 1:50 IHC)	DSHB	Cat# MF 20, RRID:AB_2147781
MEF2C (D80C1) (1:800 WB; 1:250 IF)	CST	Cat# 5030, RRID:AB_10548759
MEF2A (1:800 WB)	Abcam	Cat# AB76063, RRID:AB_1310444
MyoD (M-318) (1:500 WB; 2μg ChIP)	Santa Cruz	Cat# sc-760, RRID:AB_2148870
MEF2D (1:800 WB)	Abcam	Cat# ab32845, RRID:AB_776269
p21Waf1/Cip1 (12D1) (1:800 WB)	CST	Cat# 2947, RRID:AB_823586
MYOG (1:250 WB)	DSHB	Cat# F5D, RRID:AB_2146602

MYOG (1: 800 WB)	Santa Cruz	Cat# SC12732, RRID:AB_627980
H3K27Ac (7μg ChIP)	Active motif	Cat# 39133, RRID:AB_2561016
ERK (1:800 WB)	CST	Cat# 9102, RRID:AB_330744
Phospho-ERK (1:800 WB)	CST	Cat# 4370, RRID:AB_2315112
GAPDH (1:1000 WB)	CST	Cat# 2118, RRID:AB_561053
LaminB1 (1:5000 WB)	Abcam	Cat# ab16048, RRID:AB_10107828
αTubulin (DM1A) (1:5000 WB)	Abcam	Cat# ab7291, RRID:AB_2241126
Actin (1:1000 WB)	GenScript	Cat# A00702, RRID:AB_914102
Vinculin (hVIN-1) (1:2000 WB)	Sigma	Cat# V9131, RRID:AB_477629
HRP (Horseradishperoxidase) anti-rabbit (1:10000 WB)	CST	Cat# 7074, RRID:AB_2099233
HRP anti-mouse (1:10000 WB)	GE Healthcare	Cat# NA931, RRID:AB_772210
Goat anti mouse Alexa 488 (1:1000 IF)	Thermofisher	Cat# A28175, RRID:AB_2536161
Goat anti rabbit Alexa 568 (1:1000 IF)	Thermofisher	Cat# A-11011, RRID:AB_143157
Rabbit (DA1E) Isotype Control (1:100 IHC)	CST	Cat# 3900, RRID:AB_1550038
Drosophila Spike-in (2μg ChIP)	Active motif	Cat# 61686, RRID:AB_2737370
**Chemicals, Peptides, and Recombinant Proteins**		
Trametinib (GSK1120212)	Selleckchem	S2673
Vincristine	Hospira	NDC 61703-309-16
Drosophila Spike-in Chromatin	Active motif	53083
**BiologicalSamples**		
Tumor tissue from patients diagnosed with Fusion negative and fusion positive RMS	Bambino Gesu' Children's Hospital, IRCCS, Rome, Italy	NA

Tumor tissue from patients diagnosed with Fusion negative RMS	University of Washington, WA, USA	NA
**Critical Commercial Assays**		
ChIP-IT high sensitivity	Active motif	53040
Re-ChIP-IT	Active motif	53016
High-Capacity cDNA Reverse Transcription Kit	Thermo Fisher	4368814
RNeasy mini kit	Qiagen	74104
NEBNext Ultra DNA Library Prep Kit for Illumina	New EnglandBiolabs	E7645L
NEBNext Ultra RNA Library Prep Kit for Illumina	New EnglandBiolabs	E7530L
**Deposited Data**		
SNAI2 ChIP-seq RD, SMS-CTR, JR1		GEO GSE137168
MYOD ChIP-seq RD, SMS-CTR, JR1		GEO GSE137168
H3k27Ac ChIP-seq RD, SMS-CTR, JR1		GEO GSE137168
RNA-seq RD, SMS-CTR, JR1		GEO GSE137168
**Oligonucleotides and TaqManAssays**		
GAPDH_Forward		GGTGGTCTCCTCTGACTTCAACA
GAPDH_Reverse		GTTGCTGTAGCCAAATTCGTTGT
MCADHERIN_Forward		GGAGGACCAGGACGCCTACGA
MCADHERIN_Reverse		AGGCTGTCCGGCCTCTGTGT
MEF2C_Forward		TTCAACGCTGGACGAAGTAA
MEF2C_Reverse		AATTCCTGCATTCGTTCCTG
MEF2D_Forward		TCTTTGCCGTGACAACACC
MEF2D_Reverse		TCTCGGCACACCTTACACTG
MYOD1_Forward		CGCGACGTAGACCTGACGGC
MYOD1_Reverse		GTGGTCTTGCGCTTGCACGC
MYOGENIN_Forward		CCTGCCGTGGGCGTGTAAGG
MYOGENIN_Reverse		GGACTGCAGGAGGCGCTGTG
MHCb_Forward		AGACGGAGGAGGACAGGAAA
MHCb_Reverse		AGATCAAGATGTGGCAAAGCTAC

CDKN1A_Forward		TGTCCGTCAGAACCCATGC
CDKN1A_Reverse		AAAGTCGAAGTTCCATCGCTC
PAX7_Forward		TGTGACCGAAGCACTGTGCCC
PAX7_Reverse		AGCCGGTTCCCTTTGTCGCC
c-SNAI2_Forward		GAACTGGACACACATACAGTGATT
c-SNAI2_Reverse		GAGAGAGGCCATTGGGTAGC
SNAI2_Forward		CAGACCCTGGTTGCTTCAA
SNAI2_Reverse		TGACCTGTCTGCAAATGCTC
SNAI2_Forward_SYBR	used in Fig. 1i	TCGGACCCACACATTACCTTG
SNAI2_Reverse_SYBR	used in Fig. 1i	AAAAGGCTTCTCCCCCGTGT
GAPDH_Forward_SYBR	used in Fig. 1i	CCACCCATGGCAAATTCCATGGCA
GAPDH_Reverse_SYBR	used in Fig. 1i	CGCCCCACTTGATTTTGG
SNAI2_ MYOD_at_MYOG_SE1_Forward	used in Fig. 4	GTGTAGTCCCTTGCACTGAG
SNAI2_ MYOD_at_MYOG_SE1_Reverse	used in Fig. 4	ATTCACACTCTGACCCTGGA
SNAI2_MYOD_at_MYOG_SE2_Forward	used in Fig. 4	GACTGAAACCCGATCAGCTC
SNAI2_ MYOD_at_MYOG_SE2_Reverse	used in Fig. 4	CTGCCATCCCCATTGCTAAT
MYOD_at_MYOG_promoter_Forward	used in Fig. 4	GCCGTCGGGTGTAATTTGAT
MYOD_at_MYOG_promoter_Reverse	used in Fig. 4	GCAGATGAGAGGGGAATGTG
SNAI2_at_MRPL9_Forward	used in Fig. 4	GCCGTCGGGTGTAATTTGAT
SNAI2_at_MRPL9_Reverse	used in Fig. 4	GCAGATGAGAGGGGAATGTG
SNAI2	AppliedBiosystems	Hs00950344_m1
CDKN1A	AppliedBiosystems	Hs00355782_m1
MYOG	AppliedBiosystems	Hs01072232_m1
MYOD1	AppliedBiosystems	Hs02330075_g1
MCK	AppliedBiosystems	Hs00176490_m1
MyH2	AppliedBiosystems	Hs00430042_m1
GAPDH	AppliedBiosystems	Hs99999905_m1
Recombinant DNA		
siSlug2 shSNAI2.1	Addgene	#10904
siSlug3 shSNAI2.2	Addgene	#10905
psPAX2	Addgene	#12260
pMD2.G	Addgene	#12259

**Software and Algorithms**		
MACS2	Zhang et al.,2009	github.com/taoliu/MACS
Samtools	Li et al., 2009	samtools.sourceforge.net
Homer	Heinz et al., 2010	homer.ucsd.edu/homer
Bedtools	Quinlan et al., 2010	github.com/arq5x/bedtools2
EDEN	Berkley et al., 2017	
Tophat	Trapnell et al., 2009	github.com/infphilo/tophat
BWA	Li H. &Durbin R. 2009	bio-bwa.sourceforge.net
FastQC	Andrew S. (2010)	bioinformatics.babraham.ac.uk/projects/fastqc
STAR	Dobin et al., 2013	github.com/alexdobin/STAR
Gene Set Enrichment Analysis (GSEA)	Subramanian et al., 2005	software.broadinstitute.org/cancer/software/genepattern/
IGV	Robinson et al., 2011	software.broadinstitute.org/software/igv
Graphpad Prism7	N/A	graphpad.com
Rstudio v3.5.1	N/A	rstudio.com/products/rstudio
gRNA (related to Fig. 1i)	gRNA location	gRNAsequence
SNAI2 E1.1	Chr8:49730895-49730914	AAGCTCAAGCCTGAAGCAGG
SNAI2 E1.2	Chr8:49730898-49730917	CTCAAGCCTGAAGCAGGTGG
SNAI2 E2.1	Chr8:49562123-49562142	GAGAGCCGGAGGCCAGCTGT
SNAI2 E2.2	Chr8:49562167-49562186	TAGAATTCAAACCACCTGCT
SNAI2 E3	Chr8:49493149-49493168	AGTGGAATCTCTTCAGCTGT
SNAI2 E4.1	Chr8:49320640-49320659	GGTGCACAGCAGGGCCAGGT
SNAI2 E4.2	Chr8:49320573-49320592	TTTATGTTGGTGTGTGCAGC
SNAI2 E5.1	Chr8:49303079-49303098	AGCTGTTCTAGATGCAGCTG
SNAI2 E5.2	Chr8:49303081-49303100	GCAGCTGTTCTAGATGCAGC
NT1	non-targeting	GCCGGCGCCGAGCCGGACTTCG
NT2	non-targeting	GAGTCGCTTCTCGATTATGGG
