# Supplementary material for: Interaction between SNAI2 and MYOD enhances oncogenesis and suppresses differentiation in Fusion Negative Rhabdomyosarcoma
Source: Nat Commun. 2021 Jan 8;12:192. doi: 10.1038/s41467-020-20386-8 (PMC7794422; doi:10.1038/s41467-020-20386-8)
Supplement: Supplementary file 3 — Description of additional Supplementary Files [file 41467_2020_20386_MOESM3_ESM.pdf]

Description of Additional Supplementary Files

File Name: Supplementary Data 1
Description: Western blot source images
