# Supplementary material for: Interaction between SNAI2 and MYOD enhances oncogenesis and suppresses differentiation in Fusion Negative Rhabdomyosarcoma
Source: Nat Commun. 2021 Jan 8;12:192. doi: 10.1038/s41467-020-20386-8 (PMC7794422; doi:10.1038/s41467-020-20386-8)
Supplement: Supplementary file 4 — Supplementary Data 1 [file 41467_2020_20386_MOESM4_ESM.zip › 234521_2_data_set_5117082_qkbrl7.pptx]

## Slide 1
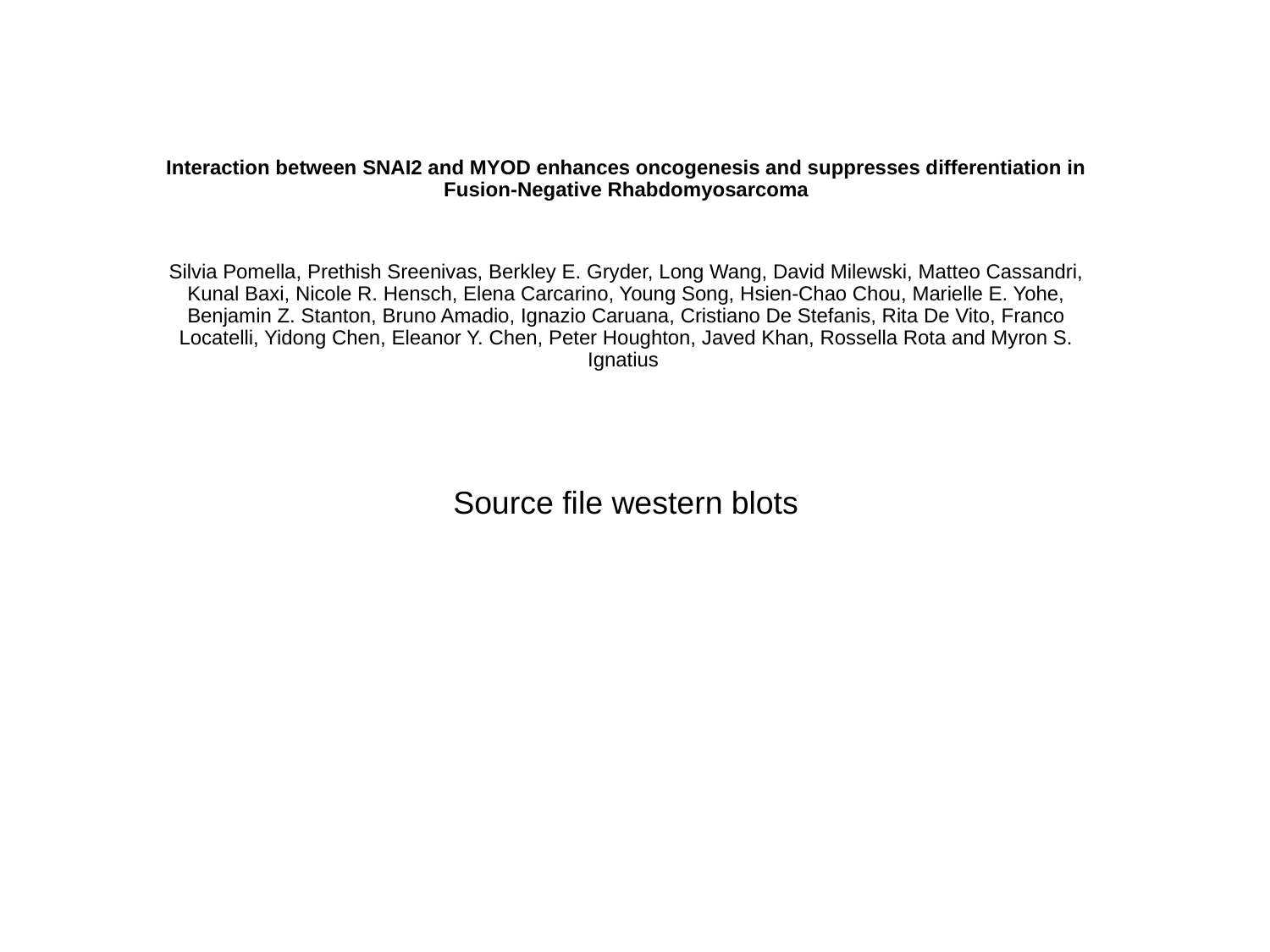

Interaction between SNAI2 and MYOD enhances oncogenesis and suppresses differentiation in Fusion-Negative Rhabdomyosarcoma
Silvia Pomella, Prethish Sreenivas, Berkley E. Gryder, Long Wang, David Milewski, Matteo Cassandri, Kunal Baxi, Nicole R. Hensch, Elena Carcarino, Young Song, Hsien-Chao Chou, Marielle E. Yohe, Benjamin Z. Stanton, Bruno Amadio, Ignazio Caruana, Cristiano De Stefanis, Rita De Vito, Franco Locatelli, Yidong Chen, Eleanor Y. Chen, Peter Houghton, Javed Khan, Rossella Rota and Myron S. Ignatius
Source file western blots

## Slide 2
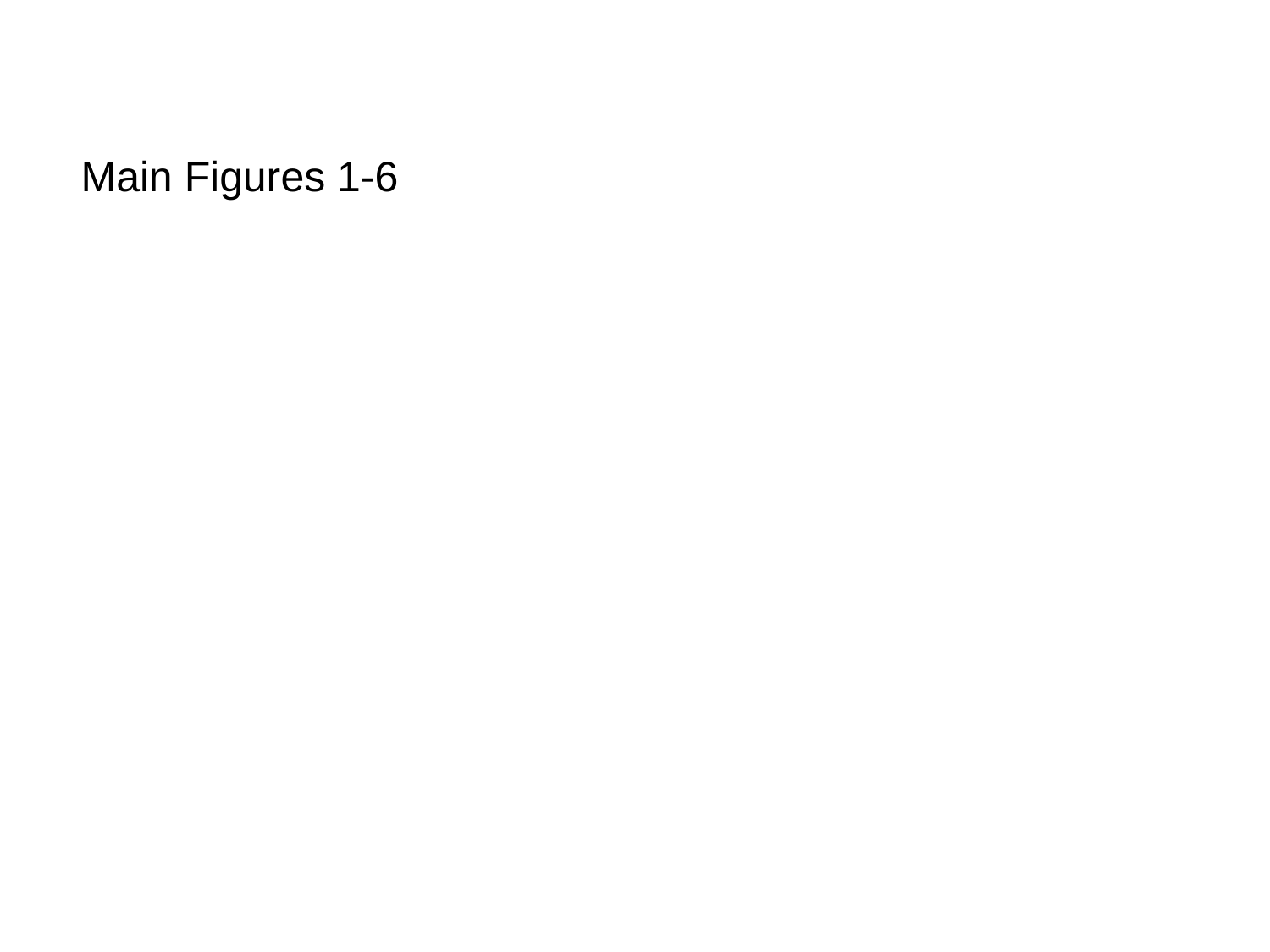

# Main Figures 1-6

## Slide 3
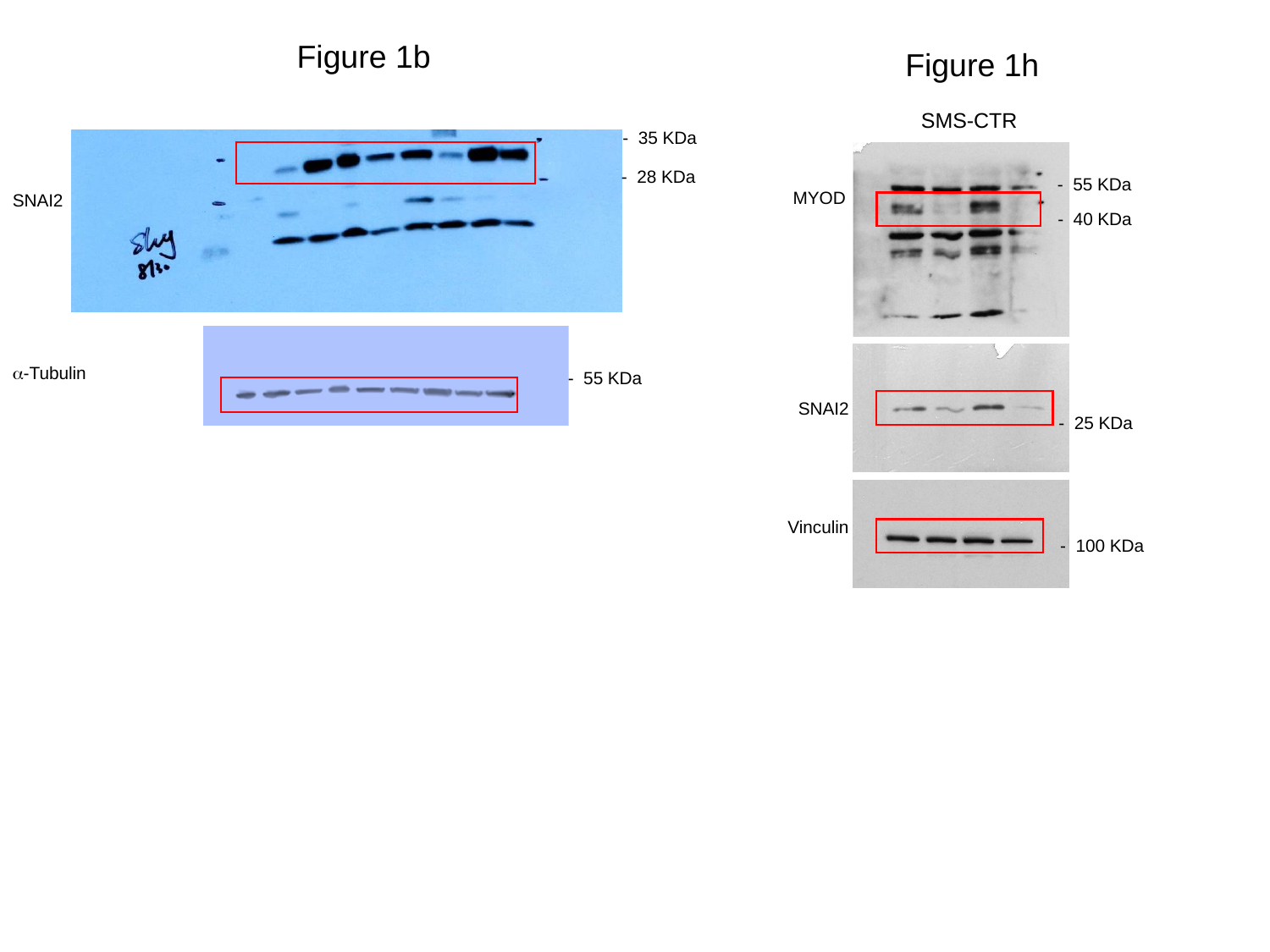

Figure 1b
SNAI2
a-Tubulin
Figure 1h
SMS-CTR
- 35 KDa
- 28 KDa
- 55 KDa
MYOD
- 40 KDa
- 55 KDa
SNAI2
- 25 KDa
Vinculin
- 100 KDa

## Slide 4
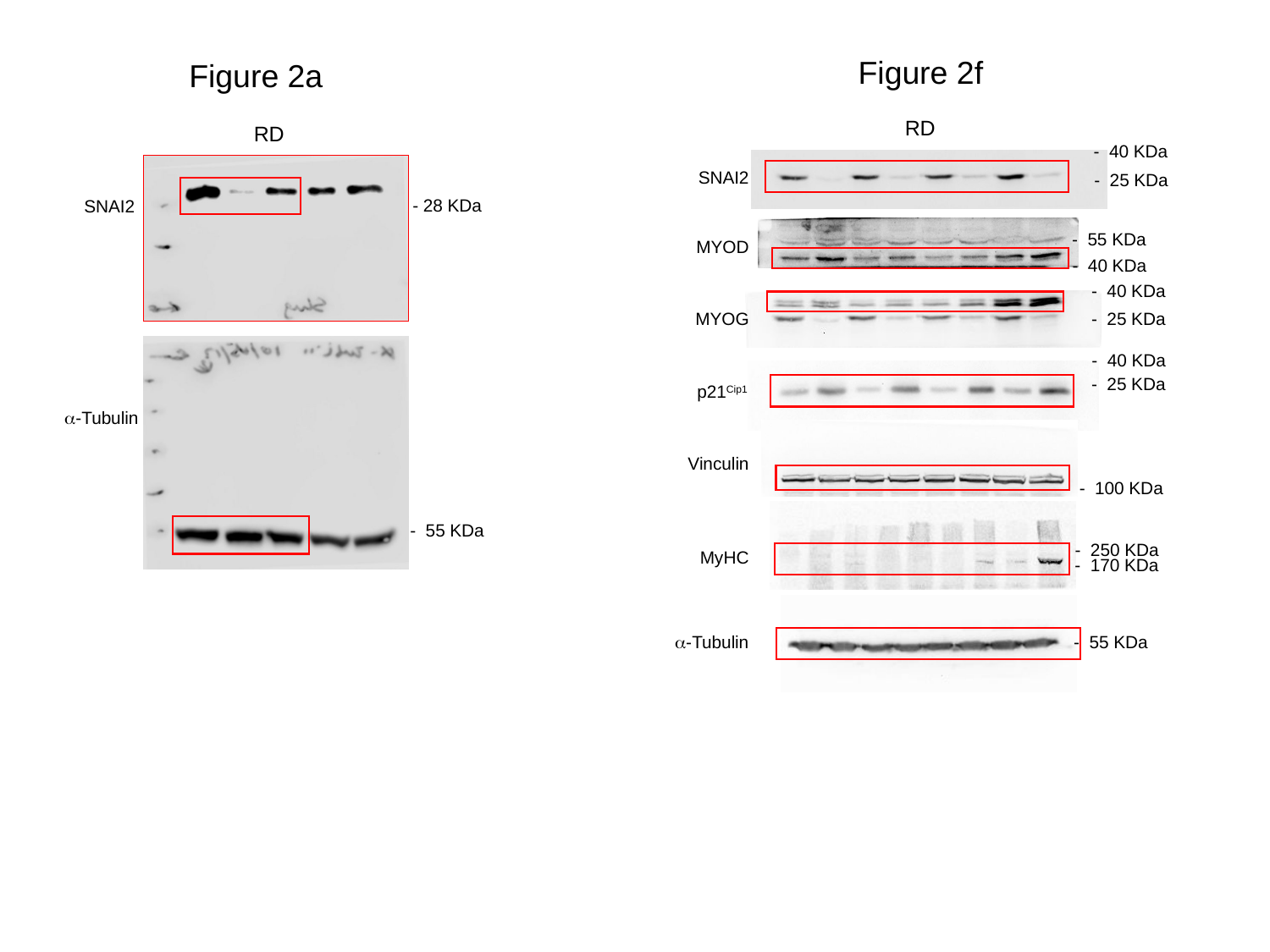

Figure 2f
 Figure 2a
 SNAI2
a-Tubulin
RD
RD
- 40 KDa
SNAI2
- 25 KDa
- 28 KDa
- 55 KDa
MYOD
- 40 KDa
- 40 KDa
- 25 KDa
MYOG
- 40 KDa
- 25 KDa
p21Cip1
Vinculin
- 100 KDa
- 55 KDa
- 250 KDa
MyHC
- 170 KDa
- 55 KDa
a-Tubulin

## Slide 5
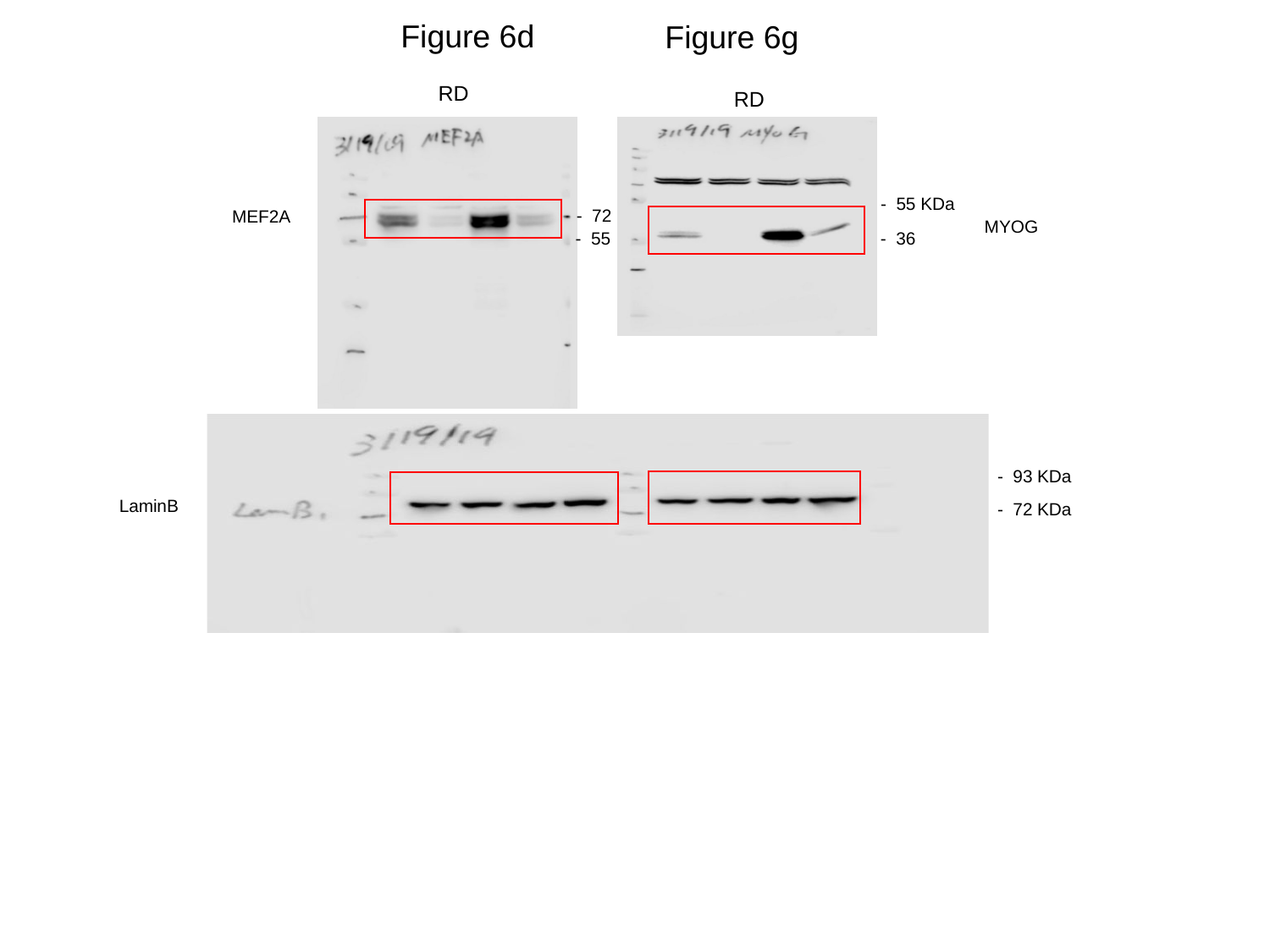

Figure 6d
Figure 6g
RD
RD
- 55 KDa
- 72
MEF2A
MYOG
- 36
- 55
- 93 KDa
LaminB
- 72 KDa

## Slide 6
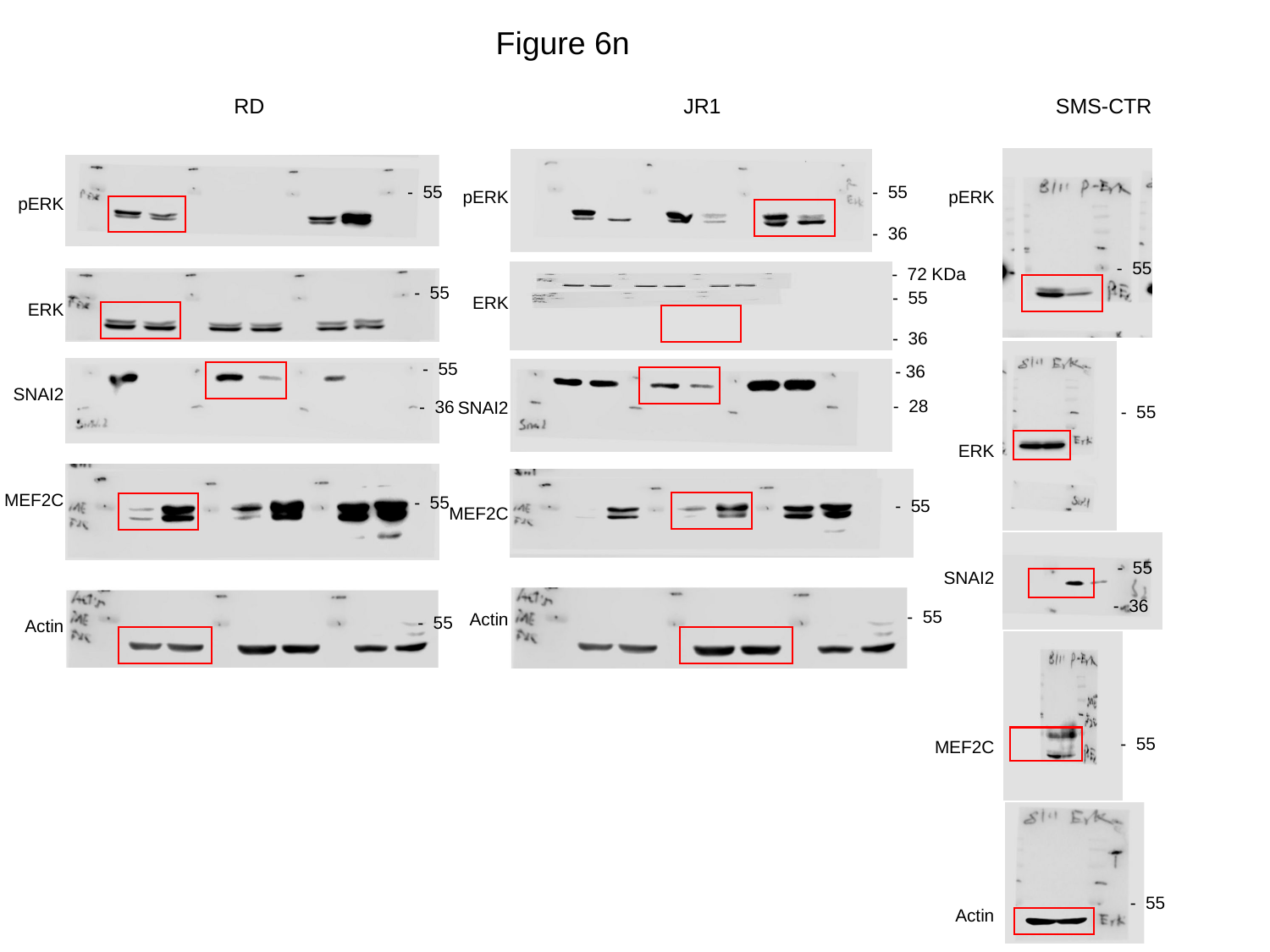

Figure 6n
RD
JR1
SMS-CTR
- 55
- 55
pERK
ERK
SNAI2
MEF2C
Actin
pERK
ERK
SNAI2
MEF2C
Actin
pERK
ERK
SNAI2
MEF2C
Actin
- 36
- 55
- 72 KDa
- 55
- 55
- 36
- 55
- 36
- 28
- 36
- 55
- 55
- 55
- 55
- 36
- 55
- 55
- 55
- 55

## Slide 7
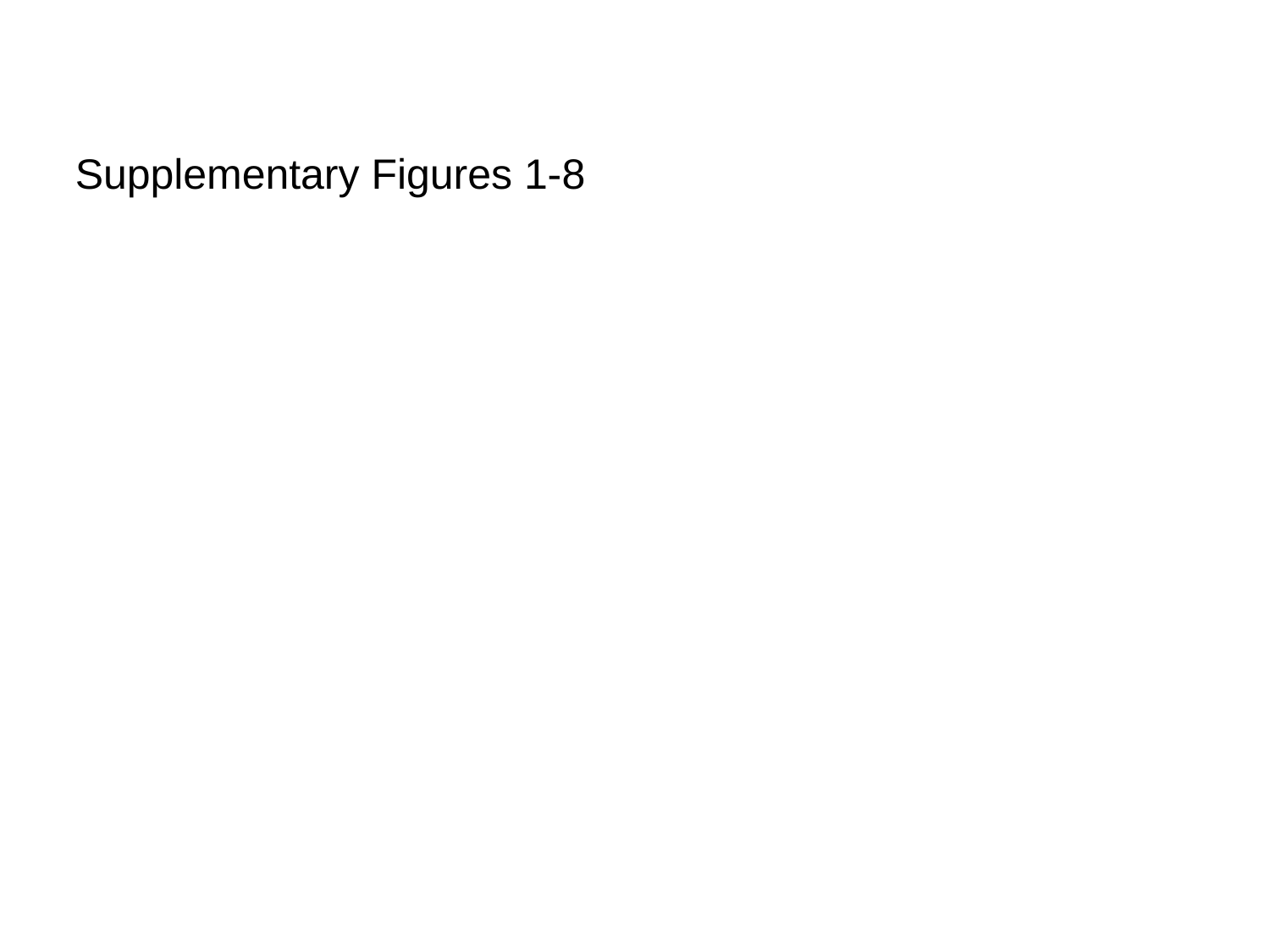

# Supplementary Figures 1-8

## Slide 8
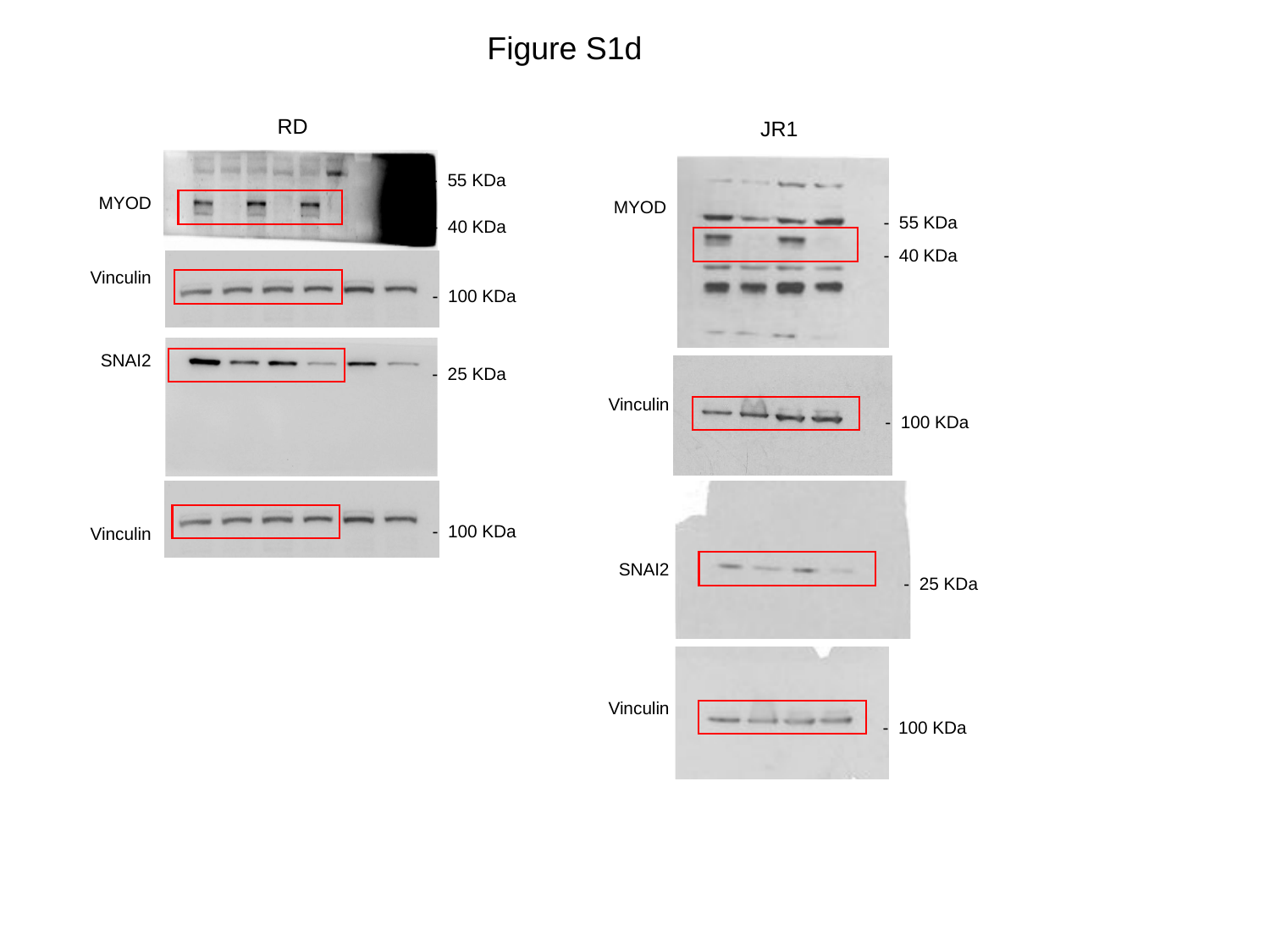

Figure S1d
RD
JR1
- 55 KDa
MYOD
MYOD
- 55 KDa
- 40 KDa
- 40 KDa
Vinculin
- 100 KDa
SNAI2
- 25 KDa
Vinculin
- 100 KDa
- 100 KDa
Vinculin
SNAI2
- 25 KDa
Vinculin
- 100 KDa

## Slide 9
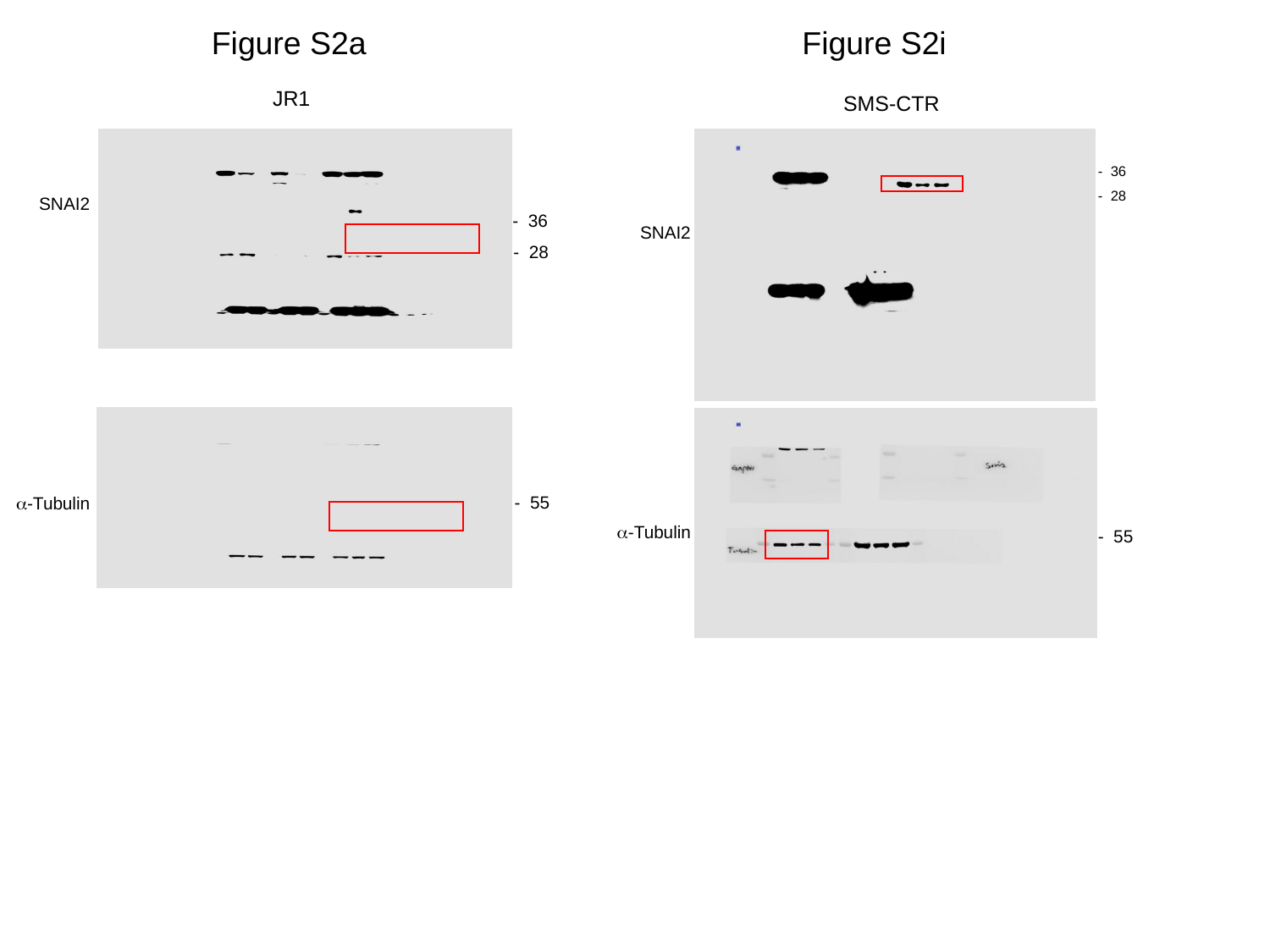

Figure S2a
Figure S2i
JR1
SMS-CTR
- 36
- 28
SNAI2
a-Tubulin
- 36
SNAI2
a-Tubulin
- 28
- 55
- 55

## Slide 10
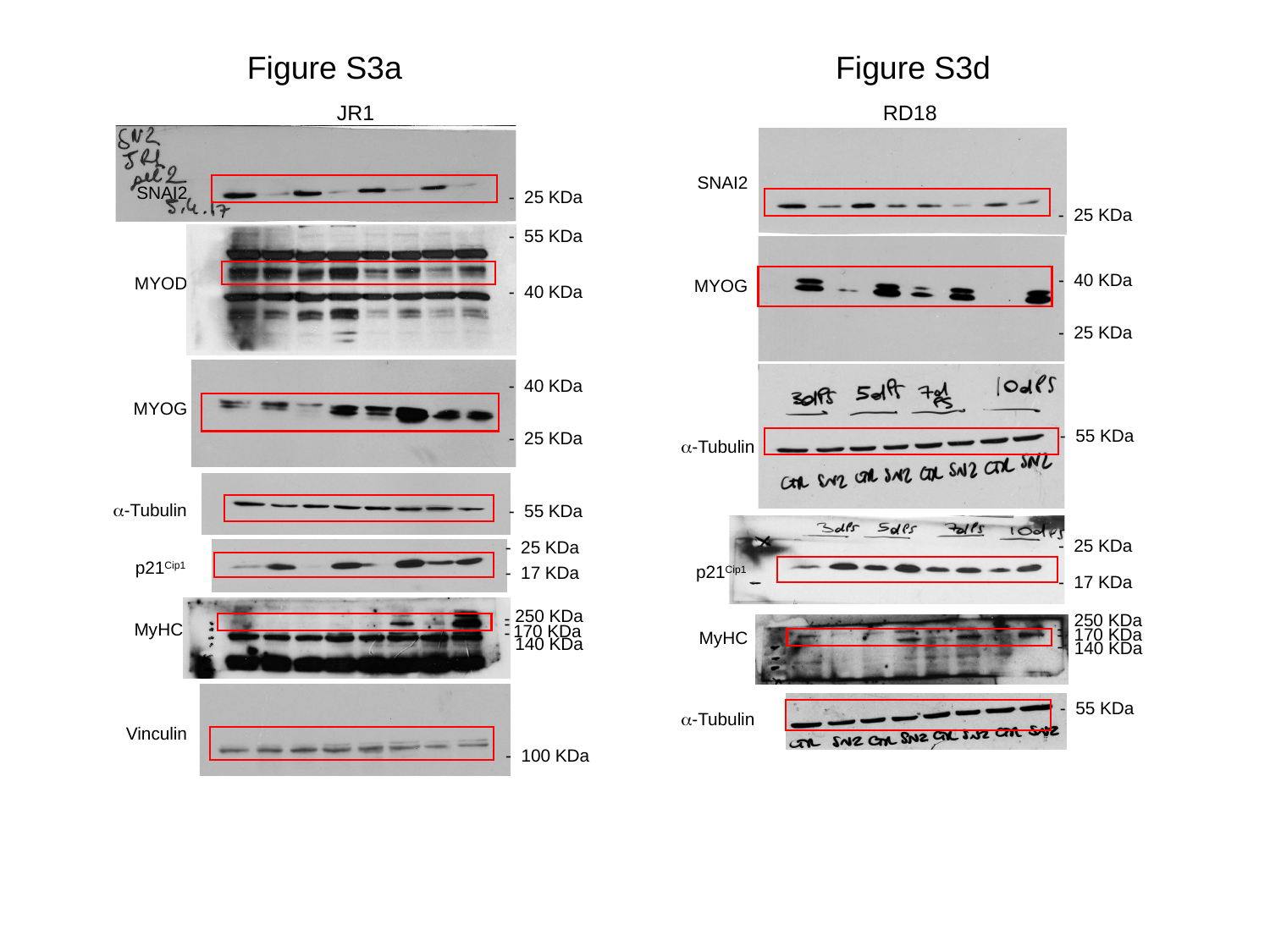

Figure S3a
Figure S3d
JR1
RD18
SNAI2
SNAI2
- 25 KDa
- 25 KDa
- 55 KDa
- 40 KDa
MYOD
MYOG
- 40 KDa
- 25 KDa
- 40 KDa
MYOG
- 55 KDa
- 25 KDa
a-Tubulin
a-Tubulin
- 55 KDa
- 25 KDa
- 25 KDa
p21Cip1
p21Cip1
- 17 KDa
- 17 KDa
250 KDa
 -
250 KDa
 -
 -
MyHC
170 KDa
 -
170 KDa
 -
MyHC
140 KDa
 -
140 KDa
- 55 KDa
a-Tubulin
Vinculin
- 100 KDa

## Slide 11
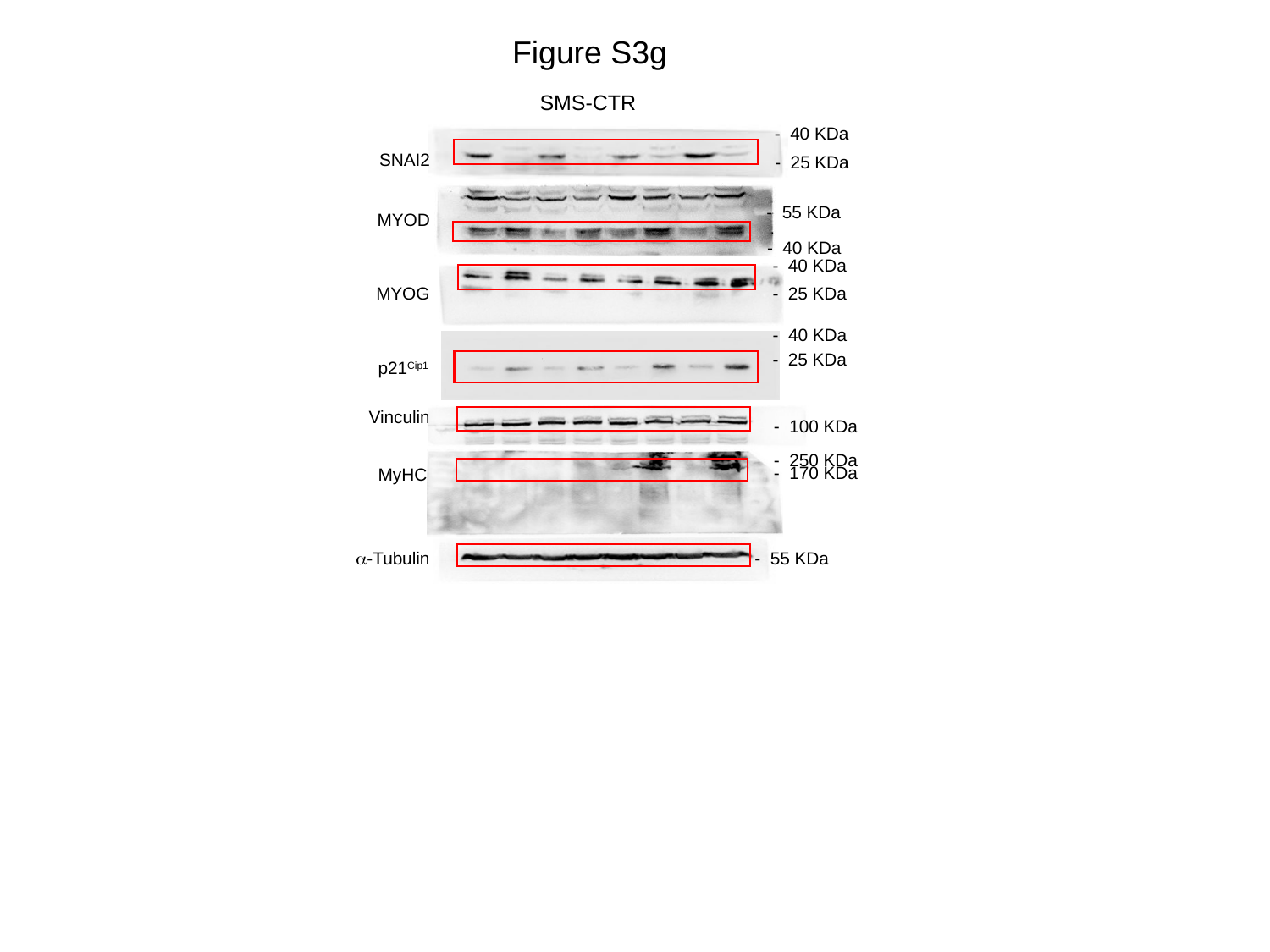

Figure S3g
SMS-CTR
- 40 KDa
SNAI2
- 25 KDa
- 55 KDa
MYOD
- 40 KDa
- 40 KDa
- 25 KDa
MYOG
- 40 KDa
- 25 KDa
p21Cip1
Vinculin
- 100 KDa
- 250 KDa
- 170 KDa
MyHC
- 55 KDa
a-Tubulin

## Slide 12
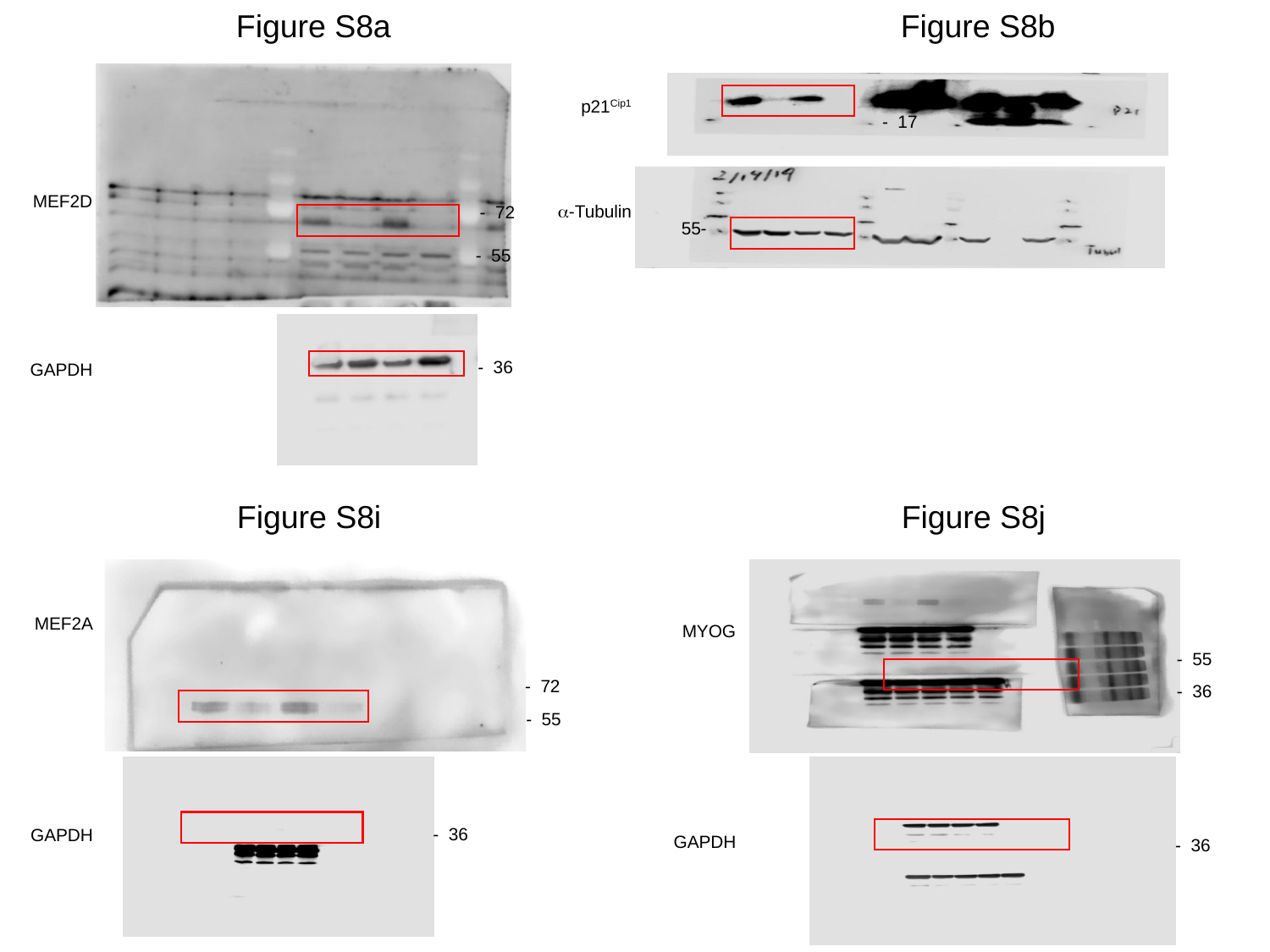

Figure S8a
Figure S8b
p21Cip1
a-Tubulin
- 17
MEF2D
GAPDH
- 72
55-
- 55
- 36
Figure S8i
Figure S8j
MEF2A
GAPDH
MYOG
GAPDH
- 55
- 72
- 36
- 55
- 36
- 36
