# Supplementary material for: Interaction between SNAI2 and MYOD enhances oncogenesis and suppresses differentiation in Fusion Negative Rhabdomyosarcoma
Source: Nat Commun. 2021 Jan 8;12:192. doi: 10.1038/s41467-020-20386-8 (PMC7794422; doi:10.1038/s41467-020-20386-8)
Supplement: Supplementary file 5 — Reporting summary [file 41467_2020_20386_MOESM5_ESM.pdf]

nature research

Corresponding author(s): Khan J, Rota R, Ignatius MS

Last updated by author(s): Nov 24, 2020

Reporting Summary

Nature Research wishes to improve the reproducibility of the work that we publish. This form provides structure for consistency and transparency in reporting. For further information on Nature Research policies, see our Editorial Policies and the Editorial Policy Checklist.

Statistics

For all statistical analyses, confirm that the following items are present in the figure legend, table legend, main text, or Methods section.

n/a	Confirmed	
☐	☒	The exact sample size (*n*) for each experimental group/condition, given as a discrete number and unit of measurement
☐	☒	A statement on whether measurements were taken from distinct samples or whether the same sample was measured repeatedly
☐	☒	The statistical test(s) used AND whether they are one- or two-sided<br>*Only common tests should be described solely by name; describe more complex techniques in the Methods section.*
☐	☒	A description of all covariates tested
☐	☒	A description of any assumptions or corrections, such as tests of normality and adjustment for multiple comparisons
☐	☒	A full description of the statistical parameters including central tendency (e.g. means) or other basic estimates (e.g. regression coefficient) AND variation (e.g. standard deviation) or associated estimates of uncertainty (e.g. confidence intervals)
☐	☒	For null hypothesis testing, the test statistic (e.g. *F*, *t*, *r*) with confidence intervals, effect sizes, degrees of freedom and *P* value noted<br>*Give P values as exact values whenever suitable.*
☒	☐	For Bayesian analysis, information on the choice of priors and Markov chain Monte Carlo settings
☒	☐	For hierarchical and complex designs, identification of the appropriate level for tests and full reporting of outcomes
☐	☒	Estimates of effect sizes (e.g. Cohen's *d*, Pearson's *r*), indicating how they were calculated

Our web collection on statistics for biologists contains articles on many of the points above.

Software and code

Policy information about availability of computer code

Data collection	Fluorescence image acquisition software Olympus FV315S-SW Version 2.4.1.198; spheres counting and image acquisition by the Celigo Imaging Cytometer automated cell counting apparatus (Nexcelom Bioscience LLC, Lawrence, MA); the light microscopy imaging was performed on a Nikon E600 light microscope equipped with NIS Elements BR software Version 4.5000.1117.0

Data analysis	Poly-A selected RNA libraries were prepared and sequenced on Illumina HiSeq2000. QC was performed using FastQC version 0.11.2 and Picard's version 1.127 RNASeqMetrics function with the default parameters. PCR duplicates were marked using Picard's MarkDuplicates function. RNA-seq reads were aligned to the UCSC hg19 reference genome using TopHat version 2.0.13. Significance was defined as having FDR q=<0.01 and FWER p=of <0.05.<br>Gene set enrichment analysis (http://www.broadinstitute.org/gsea/index.jsp) was performed using default parameter settings.<br>ChIP-seq libraries were prepared using Illumina TruSeqChIP Library Prep Kit (Illumina). Libraries were multiplexed and sequenced using the NextSeq500 (Illumina). Reads were mapped to reference genome (version hg19) using BWA. High-confidence ChIP-seq peaks were called by MACS2. Gene ontology was performed using GREAT, using hg19 and the whole genome as the background. Chromatin states were characterized using ChromHMM (http://compbio.mit.edu/ChromHMM/). Enhancers were identified using the ROSE2 (https://github.com/linlabbcm/rose2) software. Differential peak calling was performed using BEDTools v2.25.0 in multicov mode to measure read counts, which were normalized per million mapped reads, and visualized using R package ggplot2 or NGS plot. Enhancer regions were linked to their nearest gene, irrespective of strand specificity and gene direction, within topologically associated domain (TAD) boundaries using EDEN. Enrichment of known and de-novo motifs were found using HOMER. Enrichment peaks were visualized with epigenome browser and IGV.<br>HiChIP libraries were paired-end sequenced to a shallow depth of 80-120 million reads. Two independent biological replicates were combined informatically to get contact maps with 30 million valid, long-range cis contacts. Analysis was performed using HiC-Pro and visualized in Juicebox.<br>Software used for statistical tests include R (3.4.4), Graphpad Prism(8.4.2), ImageJ(2.0.0), Adobe photoshop (20.0.10), Adobe Illustrator (23.1.1) and Microsoft Excel(16.16.27).

For manuscripts utilizing custom algorithms or software that are central to the research but not yet described in published literature, software must be made available to editors and reviewers. We strongly encourage code deposition in a community repository (e.g. GitHub). See the Nature Research guidelines for submitting code & software for further information.

Data

Policy information about availability of data

All manuscripts must include a data availability statement. This statement should provide the following information, where applicable:

- Accession codes, unique identifiers, or web links for publicly available datasets
- A list of figures that have associated raw data
- A description of any restrictions on data availability

The datasets generated during this study (RNA-seq, ChIP-seq and Hi-seq) are available at GEO (Gene Expression Omnibus) with the following accession number (GSE137168). Previously published GEO datasets used in the study were: ChIP-seq in human myoblasts and myotubes (GSE29611, GSE50413), ChIP-seq in RMS and ChIP-seq and RNA-seq in Fibroblasts and Fibroblasts + PAX3-FOXO1 (GSE83728), RNA-seq in Fibroblasts and Fibroblasts + MYOD1 (GSE93263), Hi-C in IMR90 (GSE63525), ChIP-seq and RNA-seq in SMS-CTR ± Trametinib (GSE85171). A source data file accompanies this manuscript. The remaining data are available within the article, supplementary Information or available from the authors upon request.

Field-specific reporting

Please select the one below that is the best fit for your research. If you are not sure, read the appropriate sections before making your selection.

☒ Life sciences ☐ Behavioural & social sciences ☐ Ecological, evolutionary & environmental sciences

For a reference copy of the document with all sections, see nature.com/documents/nr-reporting-summary-flat.pdf

Life sciences study design

All studies must disclose on these points even when the disclosure is negative.

Sample size	In vivo studies were designed to obtain statistically significant results while maintaining minimum animal sacrifice (Arfin & Zahiruddin 2017) and signal to noise ratio of cell line experiments by power analysis.<br>No sample-size calculation was performed for the in vitro experiments. Each condition was analyzed with at least 3 biological replicates. This is typically the standard for the experiments performed so that a reasonable range of variability between samples can be accounted for.
Data exclusions	No data was excluded from the study
Replication	All attempts at replication were successful, experiments were repeated in three biological replicates unless stated otherwise
Randomization	Not relevant to the study as the cell lines and mouse used in the study were isogenic and maintained at minimally perturbative conditions.
Blinding	Blinding was not possible as the tumor phenotype was visually prominent in the shSNAI2 knock down injected tumors. For cell line experiments blinding was either not necessary as the collection and analysis were performed by software/equipment uniformly post treatment and not possible in cases were the phenotype was prominent during data collection.

Reporting for specific materials, systems and methods

We require information from authors about some types of materials, experimental systems and methods used in many studies. Here, indicate whether each material, system or method listed is relevant to your study. If you are not sure if a list item applies to your research, read the appropriate section before selecting a response.

Materials & experimental systems

n/a	Involved in the study
☐	☒ Antibodies
☐	☒ Eukaryotic cell lines
☒	☐ Palaeontology and archaeology
☐	☒ Animals and other organisms
☐	☒ Human research participants
☒	☐ Clinical data
☒	☐ Dual use research of concern

Methods

n/a	Involved in the study
☐	☒ ChIP-seq
☒	☐ Flow cytometry
☒	☐ MRI-based neuroimaging

Antibodies

Antibodies used

Slug (C19G7) CST Cat# 9585, RRID:AB_2239535
Myosin Heavy Chain DSHB Cat# MF 20, RRID:AB_2147781
MEF2C (D80C1) CST Cat# 5030, RRID:AB_10548759
MEF2A CST Cat# 9736, RRID:AB_10691852
MyoD (M-318) Santa Cruz Cat# sc-760, RRID:AB_2148870
MEF2D Abcam Cat# ab32845, RRID:AB_776269
p21Waf1/Cip1 (12D1) CST Cat# 2947, RRID:AB_823586
MYOG DSHB Cat# F5D, RRID:AB_2146602
H3K27Ac Active motif Cat# 39133, RRID:AB_2561016
ERK CST Cat# 9102, RRID:AB_330744
Goat anti mouse Alexa 488 Thermofisher Cat# A28175, RRID:AB_2536161
Goat anti rabbit Alexa 568 Thermofisher Cat# A-11011, RRID:AB_143157
Phospho-ERK CST Cat# 4370, RRID:AB_2315112
GAPDH CST Cat# 2118, RRID:AB_561053
LaminB1 Abcam Cat# ab16048, RRID:AB_10107828
aTubulin (DM1A) Abcam Cat# ab7291, RRID:AB_2241126
Vinculin (hVIN-1) Sigma Cat# V9131, RRID:AB_477629
HRP (Horseradishperoxidase) anti-rabbit CST Cat# 7074, RRID:AB_2099233
HRP anti-mouse GE Healthcare Cat# NA931, RRID:AB_772210
Rabbit (DA1E) Isotype Control CST Cat# 3900, RRID:AB_1550038
Drosophila Spike-in Active motif Cat# 61686, RRID:AB_2737370

Validation

All antibodies were validated in RMS cell line samples by cross comparing to vendor provided molecular size information using western blotting. SNAI2 antibody was validated by western blotting in SNAI2 knock down (RD,SMS-CTR,JR1) and by immunostaining in SNAI2 knock down xenografts samples and in negative control muscle tissue.

Eukaryotic cell lines

Policy information about cell lines

Cell line source(s)

RD(ATCC® CCL-136™), JR1, RH-30, RH-36 and SMS-CTR were obtained from Dr. Peter Houghton, GCCRI; RD18, from Carola Ponzetto, Dept. of Oncology, University of Turin, Italy. 293T cells (CRL-3216™) from ATCC®.

Authentication

The cell lines used were authenticated by STR profiling.

Mycoplasma contamination

Cell lines in Ignatius', Rota's and Khan's labs are tested for mycoplasma contamination by PCR every 6 months. All lines to date tested negative.

Commonly misidentified lines
(See ICLAC register)

No commonly misidentified cell lines were used in this study.

Animals and other organisms

Policy information about studies involving animals; ARRIVE guidelines recommended for reporting animal research

Laboratory animals

Mice used for xenograft were CB17 SCID females aged at 6-8 weeks. They were maintained at sterile conditions with five mice per cage and fed ad libitum. 12h light/12h dark cycle, ambient temperature 18-23°C with 40-60% humidity

Wild animals

Study did not involve wild animals.

Field-collected samples

Study did not involve field collected samples.

Ethics oversight

All mouse experiments were approved by the institutional animal welfare and ethics committees (Institutional Review Boards) at the Greehey Children’s Cancer Research Institute Accreditation of Laboratory Animal Care International (AAALAC) under protocol number

20150015AR and at the Italian Ministry of Health for the Children's Hospital Bambino Gesù/SAFU Institutes under protocol number 514/2015-PR.

Note that full information on the approval of the study protocol must also be provided in the manuscript.

Human research participants

Policy information about studies involving human research participants

Population characteristics	Formalin-fixed paraffin embedded (FFPE) tissue blocks from cases of embryonal or alveolar rhabdomyosarcoma were obtained at the diagnosis in patients aged from 1 to 19 years from department archives per Institutional Review Board approval. Biopsy of adjacent normal muscle tissues were done when ethically possible.<br>All tissue sections were de-identified prior to their use in immunohistochemical stains for the manuscript. No clinical information associated with the tissue samples was requested because the study was only aimed at evaluating the expression of the SNAI2 protein in primary tissues sections and, thus, no clinical correlations were needed or done.
Recruitment	All FFPE tissue samples were obtained retrospectively. No patients were recruited for this study.
Ethics oversight	FFPE samples were obtained from the Pathology Unit of Bambino Gesù Children's Hospital (Rome, Italy) and Department of Pathology, University of Washington (Seattle, WA) and the approval of the study was obtained from the ethics committees at the two Research Centers.<br>Authorization; IR no. 9362 for University of Washington and 120 LB, 02/10/2015 for Bambino Gesu' Children's Hospital (Rome, Italy). Written informed consent was obtained from all patients.

Note that full information on the approval of the study protocol must also be provided in the manuscript.

ChIP-seq

Data deposition

☒ Confirm that both raw and final processed data have been deposited in a public database such as GEO.

☒ Confirm that you have deposited or provided access to graph files (e.g. BED files) for the called peaks.

Data access links<br>*May remain private before publication.*	https://www.ncbi.nlm.nih.gov/geo/query/acc.cgi?acc=GSE137168
Files in database submission	JR1_shCtrl_H3K27ac_C_HLJWHBGX5<br>JR1_shCtrl_SNAI2_C_HLJWHBGX5<br>CTR_shCtrl_H3K27ac_C_H5W2CBGX7<br>CTR_shCtrl_SNAI2_C_H5W2CBGX7<br>CTR_shSNAI2_H3K27ac_C_H5W2CBGX7<br>CTR_shSNAI2_SNAI2_C_H5W2CBGX7<br>CTR_D48_SNAI2_031_CM_H3HYNBGX7<br>CTR_T48_SNAI2_031_CM_H3HYNBGX7<br>CTR_shCtrl_MYOD_035_C_HMG7VBGX7<br>CTR_shSNAI2_MYOD_035_C_HMG7VBGX7<br>RD_shCtrl_H3K27ac_037_C_HMJ2HBGX7<br>RD_shCtrl_SNAI2_037_C_HMJ2HBGX7<br>RD_shSNAI2_SNAI2_037_C_HMJ2HBGX7<br>RD_shCtrl_MYOD_037_C_HMJ2HBGX7<br>RD_shSNAI2_MYOD_037_C_HMJ2HBGX7<br>Sample_CTR_DMSO_M2_HiChIP_HCY3CBGXG_R1<br>Sample_CTR_DMSO_M2_HiChIP_HCY3CBGXG_R2<br>Sample_CTR_shScr_10d_T_UTHSCSA_R1<br>Sample_CTR_shSNAI2_10d_T_UTHSCSA_R1<br>Sample_RD_shScr_10d_T_UTHSCSA_R1<br>Sample_RD_shSNAI2_10d_T_UTHSCSA_R1
Genome browser session<br>(e.g. UCSC)	No longer applicable

Methodology

Replicates	One replicate was used for each condition.
Sequencing depth	Instrument mode: Illumina NextSeq 500 Read length: 75 Single end
Antibodies	Slug (C19G7) CST Cat# 9585, RRID:AB_2239535<br>MyoD (M-318) Santa Cruz Cat# sc-760, RRID:AB_2148870

	H3K27Ac Active motif Cat# 39133, RRID:AB_2561016<br>Drosophila Spike-in Active motif Cat# 61686, RRID:AB_2737370
Peak calling parameters	Peaks were called using MACS2 (version 2.1.1.20160309, https://github.com/taoliu/MACS) using "narrow" mode for all targets reported in this paper, as they form sharp genomic peaks. Parameters for MACS2 usage: [--format BAM --control input.bam --keep-dup all --pvalue 0.0000001]. Regions called as peaks which are known to be spurious mapping artifacts were removed before any further analysis (reference locations for sites black-listed by the ENCODE consortium, https://sites.google.com/site/anshulkundaje/projects/blacklists).<br>List of BAM files used:<br>Sample_JR1_shCtrl_H3K27ac_C_HLJWHBGX5<br>Sample_JR1_shCtrl_SNAI2_C_HLJWHBGX5<br>Sample_CTR_shCtrl_H3K27ac_C_H5W2CBGX7<br>Sample_CTR_shCtrl_SNAI2_C_H5W2CBGX7<br>Sample_CTR_shSNAI2_H3K27ac_C_H5W2CBGX7<br>Sample_CTR_shSNAI2_SNAI2_C_H5W2CBGX7<br>Sample_CTR_D48_SNAI2_031_CM_H3HYNBGX7_HGY5YBGX7<br>Sample_CTR_T48_SNAI2_031_CM_H3HYNBGX7_HGY5YBGX7<br>Sample_CTR_shCtrl_MYOD_035_C_HMG7VBGX7<br>Sample_CTR_shSNAI2_MYOD_035_C_HMG7VBGX7<br>Sample_RD_shCtrl_H3K27ac_037_C_HMJ2HBGX7<br>Sample_RD_shCtrl_SNAI2_037_C_HMJ2HBGX7<br>Sample_RD_shSNAI2_SNAI2_037_C_HMJ2HBGX7<br>Sample_RD_shCtrl_MYOD_037_C_HMJ2HBGX7<br>Sample_RD_shSNAI2_MYOD_037_C_HMJ2HBGX7
Data quality	Described in Peak calling parameters
Software	Reads were mapped to reference genome (version hg19) using BWA. High-confidence ChIP-seq peaks were called by MACS2. Gene ontology was performed using GREAT, using hg19 and the whole genome as the background. Chromatin states were characterized using ChromHMM (http://compbio.mit.edu/ChromHMM/). Enhancers were identified using the ROSE2 (https://github.com/linlabbcm/rose2) software. Differential peak calling was performed using BEDTools v2.25.0 in multicov mode to measure read counts, which were normalized per million mapped reads, and visualized using R package ggplot2 or NGS plot. Enrichment peaks were visualized with epigenome browser and IGV.
